# Diversity of Immunoglobulin Light Chain Genes in Non-Teleost Ray-Finned Fish Uncovers IgL Subdivision into Five Ancient Isotypes

**DOI:** 10.3389/fimmu.2018.01079

**Published:** 2018-05-28

**Authors:** Sergey V. Guselnikov, Konstantin O. Baranov, Alexander M. Najakshin, Ludmila V. Mechetina, Nikolai A. Chikaev, Alexey I. Makunin, Sergey V. Kulemzin, Daria A. Andreyushkova, Matthias Stöck, Sven Wuertz, Jörn Gessner, Wesley C. Warren, Manfred Schartl, Vladimir A. Trifonov, Alexander V. Taranin

**Affiliations:** ^1^Laboratory of Immunogenetics, Institute of Molecular and Cellular Biology SB RAS, Novosibirsk, Russia; ^2^Novosibirsk State University, Novosibirsk, Russia; ^3^Laboratory of Comparative Genomics, Department of the Diversity and Evolution of Genomes, Institute of Molecular and Cellular Biology SB RAS, Novosibirsk, Russia; ^4^Leibniz-Institute of Freshwater Ecology and Inland Fisheries, Berlin, Germany; ^5^School of Medicine, McDonnell Genome Institute, Washington University, St. Louis, MO, United States; ^6^Department of Physiological Chemistry, Biocenter, University of Würzburg, Würzburg, Germany; ^7^Department of Biology, Hagler Institute for Advanced Study, Texas A&M University, College Station, TX, United States; ^8^Comprehensive Cancer Center Mainfranken, University Hospital Würzburg, Würzburg, Germany

**Keywords:** *Acipenser ruthenus*, Acipenseriformes, Polypteriformes, Holostei, evolution, immunoglobulin light chain

## Abstract

The aim of this study was to fill important gaps in the evolutionary history of immunoglobulins by examining the structure and diversity of IgL genes in non-teleost ray-finned fish. First, based on the bioinformatic analysis of recent transcriptomic and genomic resources, we experimentally characterized the IgL genes in the chondrostean fish, *Acipenser ruthenus* (sterlet). We show that this species has three loci encoding IgL kappa-like chains with a translocon-type gene organization and a single VJC cluster, encoding homogeneous lambda-like light chain. In addition, sterlet possesses sigma-like VL and J-CL genes, which are transcribed separately and both encode protein products with cleavable leader peptides. The Acipenseriformes IgL dataset was extended by the sequences mined in the databases of species belonging to other non-teleost lineages of ray-finned fish: Holostei and Polypteriformes. Inclusion of these new data into phylogenetic analysis showed a clear subdivision of IgL chains into five groups. The isotype described previously as the teleostean IgL lambda turned out to be a kappa and lambda chain paralog that emerged before the radiation of ray-finned fish. We designate this isotype as lambda-2. The phylogeny also showed that sigma-2 IgL chains initially regarded as specific for cartilaginous fish are present in holosteans, polypterids, and even in turtles. We conclude that there were five ancient IgL isotypes, which evolved differentially in various lineages of jawed vertebrates.

## Introduction

Immunoglobulins (Ig) are heteromeric glycoproteins that play a crucial role in the humoral immune defense of all jawed vertebrates. The Ig molecules are generally composed of heavy (IgH) and light (IgL) chains. During phylogeny, multiple isotypes of both IgH and IgL chains have evolved (1–4). The IgH isotypes, also known as classes, usually have specialized effector and/or transport functions. Their class-characteristic tertiary structure and specific determinants provide the differential binding of antibodies to Fc receptors, components of the complement system, and transport receptors on mucosal surfaces.

In contrast to IgH, the functional specialization of IgL isotypes is still poorly understood. Two isotypes—κ (kappa) and λ (lambda)—have been originally identified in mammals. Birds and squamate reptiles turned out to possess only λ chains (except for Iguanidae lizards also having κ isotypes) (5). A number of isotypes were described in frogs (6–8), teleost fish (9–12), and cartilaginous fish (13–15). However, because of the gaps in the available data, the relationships of IgLs found in different vertebrate lineages remained controversial for a long time. It was only after the identification of sigma (σ)-like chains in the nurse shark, when Criscitiello and Flajnik (16) categorized IgLs into four main isotypes: κ, λ, σ, and σ-2 (sigma-2, originally sigma-cart). To date, all four isotypes have been found only in sharks and in coelacanth [reviewed in Ref. (17)].

According to the latter classification, the teleost IgLs have been subdivided into σ-like (previous L2) and two groups of κ-like chains (L1/κG and L3/κF). Most recently, one more IgL isotype encoded by 1–2 VJC clusters was described in catfish, cod, and trout (11, 18). The isotype was suggested to be the teleostean ortholog of λ IgLs because of the λ-like organization of the recombination signal sequences (RSS). However, both V and C regions of teleostean “λ” showed only weak similarity to shark, coelacanth, and tetrapod λ chains. Also no solid evidence in favor of λ orthology was provided by phylogenetic analysis.

We reasoned that the evolutionary history of the IgL genes may be clarified by their examination in species representing non-teleost lineages of ray-finned fish (Actinopterygii), such as the Acipenseriformes (sturgeons and paddlefish), Polypteriformes (bichirs and ropefish), and Holostei (gars and bowfin). It is now generally accepted that Polypteriformes is the basal lineage of ray-finned fish, Acipenseriformes occupies an intermediate position, and Holostei is a sister group to Teleostei (19–21).

Thus far, the studies of Ig in non-teleost ray-finned fish were mainly focused on IgH loci (22–26). The IgL gene structure was explored only in Acipenseridae. Two decades ago, the Siberian sturgeon was shown to have a large family of IgL κ-like genes organized in a translocon manner (27, 28). The family was suggested to include at least 2 C genes, a group of J-segments and more than 70 V-genes. The most recent study of the Chinese sturgeon transcriptome also reported the presence of the λ-like IgL genes in this species (26). However, no detailed information on the structure, phylogeny, and diversity of these genes has been presented. It has also remained unknown if non-teleost ray-finned fish may possess σ or σ-2 chains.

In this study, we performed bioinformatic analysis of recent transcriptomic and/or genomic resources for four Acipenseriformes species: sterlet (*Acipenser ruthenus*), Siberian sturgeon (*Acipenser baerii*), Chinese sturgeon (*Acipenser sinensis*), and American paddlefish (*Polyodon spathula*). The results were used for a detailed experimental characterization of the IgL genes in sterlet. This species was shown to possess three loci of genes for IgL κ-like chains, a single Ig λ-like VJC cluster, as well as one σ-like V and one σ-like C gene. The data obtained from Acipenseriformes were extended by the bioinformatic identification of IgL genes in a holostean spotted gar (*Lepisosteus oculatus*) and in two polypterid species, saddled bichir (*Polypterus endlicheri*), and ropefish (*Erpetoichthys calabaricus*). The inclusion of IgL sequences from non-teleost ray-finned fish into the phylogenetic analysis showed a clear subdivision of IgL chains into five groups. The teleostean IgL “λ” turned out to be a κ and λ chain paralog that emerged before the radiation of ray-finned fish but has been lost in Acipenseriformes. Assuming the λ-like organization of RSS in the genes encoding this isotype, we designate it λ-2. Therefore, our data suggest that the diversity of IgL chains in various lineages of jawed vertebrates is a result of differential evolution of five ancient IgL isotypes.

## Materials and Methods

### Animals

Sterlets (*A. ruthenus*) were either caught by trawling in the Ob river near Novosibirsk (specimens A1–20), or obtained from an aquaculture farm in Russia (closely related specimens B1 and B2 originated from the Ob-Irtysh basin) and from a commercial breeder in Germany (specimen D). Siberian sturgeon (*A. baerii*) was obtained from an aquaculture farm (specimen C originated from the Yenisei river basin).

### Ethics Approval Statement

This study was conducted in accordance with the recommendations of the Animal Research Guidelines of the Ethics Committee on Animal and Human Research of the Institute of Molecular and Cellular Biology (Novosibirsk, Russia). All the protocols were approved by the Ethics Committee on Animal and Human Research of the Institute of Molecular and Cellular Biology (Novosibirsk, Russia).

### Genome and Transcriptome Sequencing and Assembly

The whole-genome DNA of B sterlet specimens was extracted using the conventional phenol–chloroform method (29). Total RNA was isolated from the spleens of the specimens B1 and C using Trizol reagent (Ambion) according to the manufacturer’s recommendations. Genomic and transcriptomic sequencing libraries were prepared and sequenced on Illumina HiSeq2000 according to the manufacturer’s protocols. All Illumina reads were trimmed with Trimmomatic (30). Draft genome assemblies were constructed with SOAPdeNovo (31). For transcriptomic data, assembly was performed with Trinity (32) (Table S1 in Supplementary Material).

### cDNA Library Construction and Screening

Poly(A)+ RNA was isolated from A1–20 specimens by chromatography on oligo(dT)-cellulose as described in Ref. (33). cDNA was prepared from leukocyte poly(A)+ using cDNA synthesis kit (Stratagene) according to the manufacturer’s instructions. The cDNA was ligated into the EcoRI-XhoI cut and dephosphorylated pBluescript SK(+) vector. After transformation into XL-1 Blue MRF’ electrocompetent cells, the unamplified library contained 2 ×10^6^ independent recombinant clones. The library was amplified and screened with V1.1- and V1.2-specific ^32^P-labeled probes as described in Ref. (29) resulting in an identification of nine cDNA clones (AF128800, AF129436-7, AF130730, AF131056, AJ133187-9, AJ236869).

### Southern Blot Analysis

Genomic DNA from sterlet blood leukocytes (A1–11 specimens) was isolated as described by Ref. (34) and digested to completion with restriction endonuclease PvuII. The digested DNA (10 μg/lane) was separated on 1% agarose gel at 30V for 14 h and transferred to a nylon membrane (Hybond-N, Amersham) using the vacuum blotting technique in 0.25 M NaOH, 1.5 M NaCl. The filters were fixed by UV cross-linking, prehybridized, and hybridized with ^32^P-labeled fragments specific to VL and CL. The following probes were used: the BamHI–BamHI fragment (1–268) of the clone ArL1B (V1-specific), the BamHI–BamHI fragment (1–266) of the clone ArL29 (V2-specific), and the EcoRV–XhoI fragment (547–1,038) of the clone ArL1B (C-specific). Prehybridization and hybridization were performed at 65°C according to the manufacturer’s instructions. The membrane was washed in 2× SSPE/0.1% SDS for 20 min twice and then in 0.2× SSC/0.1% SDS for 20 min twice at 65°C.

### Fluorescence *In Situ* Hybridization (FISH) Analysis

Optimized protocols for *A. ruthenus* cell cultivation, chromosome preparation, and FISH have been described earlier (35). IgL1-specific painting probe was generated and biotin-labeled using PCR with IgL1.03 cDNA and V1.1-specific primers (Table S2 in Supplementary Material) as described in Ref. (36).

### IgL cDNA Cloning

5′- and 3′-RACE SMART cDNA was synthesized using the Mint-2 cDNA synthesis kit (Eurogen) and 2 μg sterlet spleen total RNA (Trizol isolated from B2 specimen). For 5′-RACE cDNA synthesis, we used PlugOligo-1 adapter and plain oligo(dT) (Fermentas); for 3′-RACE—CDS-1 adapter only. PCR reactions (30–35 cycles) were carried out using RACE cDNA, universal, and/or gene-specific primers (Table S2 in Supplementary Material) and Phusion polymerase (Thermo scientific) according to the manufacturer’s recommendations. IgL cDNA amplicons were purified using AmpliClean magnetic beads (Nimagen) and ligated into pBluescript KS II (Stratagene) vector digested with EcoRV endonuclease. Insert-positive clones were obtained through blue/white *E. coli* colony screening and sequenced using BigDye 3.1 on Genetic Analyzer 3500 (Applied Biosystems). All cloned cDNAs were deposited in GenBank with the following accession numbers: MG029293-MG029354.

### Miseq Sequencing

5′-RACE cDNA was amplified using universal and CL-specific primers as described herein above and reamplified (20 cycles) using universal and nested gene-specific primers. IgL cDNA pools were purified using AmpliClean magnetic beads (Nimagen), quantified with Nanodrop 2000c (Thermo Scientific), mixed together in an equimolar ratio and used as a template for Miseq library preparation using the Illumina TrueSeq Library Prep Kit v2. The Illumina MiSeq Reagent kit v3 was used for the library sequencing (300 bp read from both ends, partial load). The obtained reads were trimmed with Trimmomatic (30), FLASh assembled (37), and sorted into a corresponding IgL pool. All cDNAs within each pool were translated and all cDNAs with frame shifts and in-frame stop codons along with truncated cDNAs were discarded. As a result, we obtained 1,198 IgL2, 293 IgL3, and 5,413 IgL4 cDNA sequences (see Data Availability Statements).

### Computational and Phylogenetic Analysis

Blastn, tblastn, and blastp searches were performed using utilities on the NCBI,^1^ Ensembl,^2^ and Fish T1K^3^ websites. American paddlefish (*P. spathula*), saddled bichir (*P. endlicheri*), and ropefish (*E. calabaricus*) transcriptomes were only available on the Fish T1K website. The datasets for the *A. sinensis* transcriptome (38), *A. ruthenus* B1, B2, D genomes, and *A. ruthenus* B2 and *A. baerii* spleen transcriptomes were blasted using Galaxy tool version 0.1.07 (39) at the IMCB computer cluster. CL1–4 reads per nucleotide counting in B1 transcriptome was performed using Genomecov package from Bedtools toolset version 2.6 (40). IgL sequences were aligned using Clustal or Muscle utilities of the MEGA6 software (41) and corrected manually. Phylogenetic analysis was performed with the MEGA6 software using nucleotide sequences after amino acid alignment. Phylogenetic trees were constructed by the Neighbor-joining (NJ) method using nucleotide sequences after amino acid alignment. The evolutionary distances were computed using the *p*-distance method and are in the units of the number of base differences per site. All positions with less than 95% site coverage were eliminated. Maximum likelihood (ML) and minimum evolution (ME) trees were essentially the same as the NJ tree in the major branching patterns.

### Data Availability Statements

Both Miseq datasets and amino acid alignments corresponding to nucleotide alignments used for phylogenetic analysis can be found in the Figshare repository.^4^ All cDNA, genomic, and transcriptomic unique sequences analyzed in this study have GenBank accession numbers or are provided in the supplementary files. Acipenseridae genomic and transcriptomic datasets for B1, B2, and C specimens were obtained in the Institute of Molecular and Cellular Biology SB RAS (Novosibirsk, Russia). The sterlet D genome assembly dataset was obtained from the sterlet (*A. ruthenus*) genome consortium including the Leibniz-Institute of Freshwater Ecology and Inland Fisheries (IGB, Berlin, Germany), the McDonnell Genome Institute, School of Medicine (Washington University, St. Louis, MO, USA), the Institute of Molecular and Cellular Biology SB RAS (Novosibirsk, Russia), and the Department of Physiological Chemistry, Biocenter (University of Würzburg, Germany). All the datasets analyzed in this article will be disclosed with the publication of the article(s) describing them. Requests to access the datasets should be directed to Dr. Vladimir Trifonov, vlad@mcb.nsc.ru.

## Results

### Identification of Four Acipenseriformes IgL Isotypes

Using our strategy for the characterization of sterlet IgL diversity (Figure S1 in Supplementary Material), we first searched Acipenseriformes transcriptomic and genomic resources (see Computational and Phylogenetic Analysis) for sequences encoding IgL constant (CL) regions and identified four distinct CLs that were similar to each other not more than 49% at the amino acid level. We hypothesized that these sequences represent distinct loci that we preliminarily called IgL1–4. The IgL1 sequences were highly homologous to the previously described Siberian sturgeon κ-like IgLs (27, 28). IgL2–IgL4 were unknown. At the next steps, the VL segments linked to the identified CLs were used as probes to search for related VL and associated CL sequences in the Acipenseriformes transcriptomes and genomes. Third, the diversity of VL sequences associated with particular CL regions was estimated by 5′-RACE amplification of sterlet spleen cDNAs using CL-specific primers with subsequent Illumina MiSeq sequencing. Apart from the information on the rearranged VL gene repertoires, the sequencing data provided a possibility to determine 5′-UTR sequences of the expressed VL genes and to design a series of corresponding primers. The primers were used to clone full-size cDNA sequences. Finally, the generated diversity of V-J junctions was analyzed on the basis of the cloned cDNAs, MiSeq sequencing data, and available genomic sequences (Figure S1 in Supplementary Material).

### IgL1

#### Diversity of VLs and CLs

Using the approach described in Section “Identification of Four Acipenseriformes IgL Isotypes,” we analyzed the sterlet IgL1 repertoire and cloned a total of 54 unique sterlet IgL1 cDNAs (Figure S2 in Supplementary Material). Of these, 45 were produced by PCR from a specimen B2 and 9 were isolated by screening a leukocyte cDNA library obtained from a group of A specimens (see Animals). The cDNA comparisons showed that 50 of them are likely derived from different V genes, as they have multiple nucleotide substitutions relative to each other and CDR1/CDR3 regions of very different lengths. We estimate that only three groups of clones in our dataset may have originated from common V genes. Of note, IgL1.03 and IgL1.15 clones have their V segments differing only by a single nucleotide substitution (yet possessing distinct J segments); clones IgL1.28 and IgL1.34 differ by three nucleotide substitutions in their CDR2; clones IgL1.29, IgL1.30, and IgL1.37 have identical CDRs but differ by several substitutions in their FR regions (Figure S2 in Supplementary Material).

**Figure 1:**
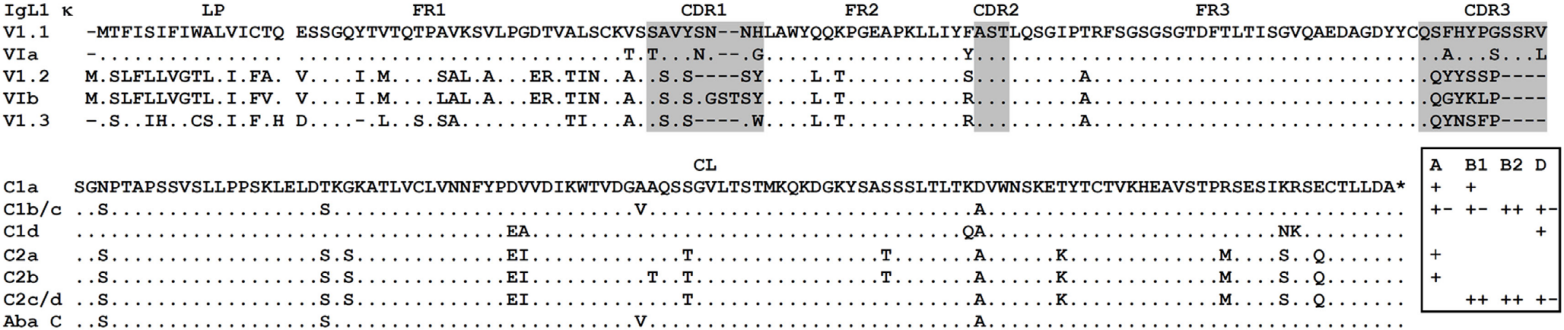
Sterlet IgL1 κ immunoglobulin light chains contain VLs of three subtypes (V1.1–V1.3) and CLs of two types (C1 and C2). Dots represent identical to consensus amino acid residues, dashes—gaps introduced for alignment, asterisk—stop codon. Leader peptide is separated from V domain by space. VL and CL amino acid sequences were deduced from following cDNAs: V1.1 (MG029295), V1.2 (MG029321), V1.3 (MG029331), C1a (AJ133187), C1b (AJ133188), C1c (MG029299), C1d (D genome, unpublished), C2a (AF129436), C2b (AF129437), C2c (MG029330), and C2d (MG029332). Siberian sturgeon sequences are provided for comparison: VIa (AJ387793), VIb (AJ387790), and C (X90557). The presence/absence (plus/minus) of different C variants **(a–d)** in different sterlet specimens **(A–D)** is shown in the frame.

**Figure 2:**
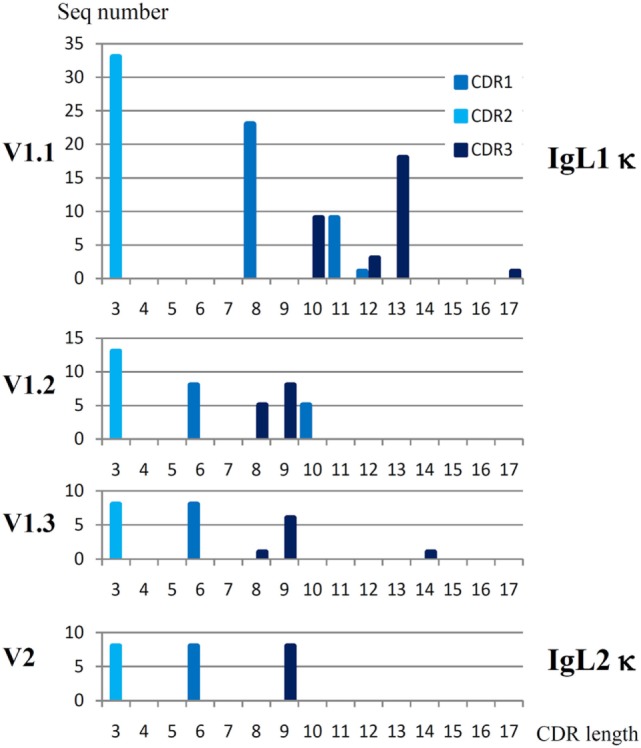
Sterlet IgL1 κ and IgL2 κ VL domains have different patterns of CDR lengths. CDR regions were determined according to the IMGT standard (42). Parent sequences may be found at the Figure S2 in Supplementary Material and Figure 6.

IgL1 V genes may be subdivided into three subfamilies, V1.1–1.3 (Figure 1) according to the 75% nucleotide identity criterion. The V1.1 and V1.2 are counterparts of the Siberian sturgeon VIa and VIb (27). The V1.3 subfamily has not been described previously. V1.3 domains are highly similar to those of V1.1 in the FR2 and FR3 framework regions, but strongly differ in the FR1 and leader peptide (LP) sequences. An estimation of the length of the CDR regions according to the IMGT standard (42) also showed that V1.1 domains differ from V1.2 and V1.3 in the length of the CDR1 and CDR3 (Figure 2).

There were 33 V1.1, 13 V1.2, and 8 V1.3 unique sequences among the cloned sterlet cDNAs (Table S3 in Supplementary Material). The Southern blot hybridization supported the existence of more than 20–30 V1.1 genes in sterlet and, more importantly, demonstrated that the V1.2 family is much smaller in size (Figure S3 in Supplementary Material). Given the fact that all cloned sterlet V1.1 cDNAs and all Siberian sturgeon VIa cDNAs were unique (Table S3 in Supplementary Material) and also taking into consideration the V1 diversity in the genomic and transcriptomic data, we suppose that the number of V1.1 genes in sterlet is around 100. Using the same criteria, we estimate the number of the V1.2 and V1.3 genes to be roughly 10–15 for each subfamily (Table 1).

**Table 1:** Sterlet IgL loci V and J segments number and recombination signal sequences (RSS) type.

No	Isotype	V1.1	V1.2	V1.3	J	C	RSS
1	IgL1A/κ1A	Up to100	Few	–	3–4	1	12/23
2	IgL1B/κ1B	10–15	10–15	10–15	6	1	12/23
		**V2.1**	**V2.2**	**V2.3**	**J**	**C**	
3	IgL2/κ2	6	1	1	2	1	12/23
			**V**		**J**	**C**	
4	IgL3/λ		1		1	1	23/12
5	IgL4/σ		–		1	1	–

The cloned IgL1 cDNAs encoded two major subtypes of CL domains, which were 90% identical to each other. We designated them as C1 and C2. Further comparison of the cDNA clones with the sterlet transcriptomic and genomic data showed that each of these C gene subtypes is represented by at least four sequence variants, differing by 2–11 nucleotide substitutions (C1a,b,c,d and C2a,b,c,d). We suggest that C1 and C2 are encoded by two distinct C genes while their variants are allelic. The latter suggestion is supported by the fact that no more than two C1 variants and no more than two C2 variants were identified in each fish specimen (Figure 1). For instance, cloned IgL1 cDNAs from sterlet B2 contain C1b and C1c as well as C2c and C2d sequences, but do not contain C1a, C1d, C2a, or C2b sequences.

The presence of two distinct CL genes of the IgL1 isotype in sterlet was supported by the results of the Southern blot hybridization (Figure S3 in Supplementary Material). Moreover, we found that C1 and C2 genes are associated with different sets of the V region genes and may represent two distinct IgL1 loci, IgL1A, and IgL1B (Table 1). C1 was found only in association with the V1.1 and V1.2 subfamilies, whereas C2 was associated with all three IgL1 VL subfamilies. When C1-specific primer was used in RT-PCR, we observed the major product with V1.1-specific primer, the minor with the V1.2, and no products with the V1.3 primer. With the C2-specific primer, V1.1-, V1.2-, and V1.3-specific primers produced PCR fragments of comparable intensity (Figure S4 in Supplementary Material; Table 1).

#### Structure of the IgL1 Loci

**Figure 3:**
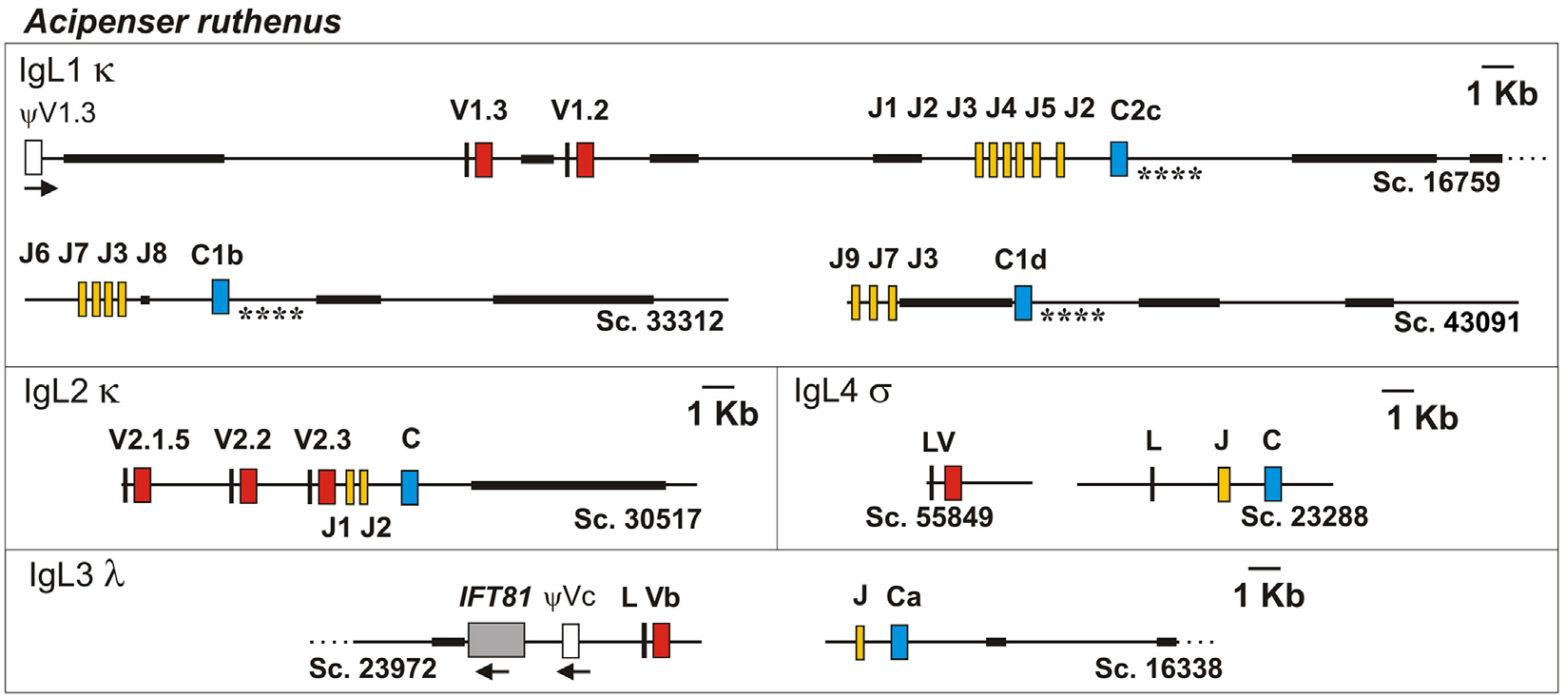
The structure of Sterlet IgL1-4 loci according to the genomic sequence analysis. Genomic scaffolds (Sc.) were extracted from sterlet D draft genome assembly. Exons encoding leader peptides (L) are denoted by vertical lines, variable (V), joining (J), and constant (C) segments are denoted by red, yellow, and blue rectangles, respectively. Pseudo genes are denoted by white rectangles. Black bold lines on scaffolds mark NNN-stretch areas. Asterisks mark compared regions of C1- and C2-containing scaffolds (see text). The transcriptional direction of all genomic elements is from the left to the right, if not shown otherwise.

**Figure 4:**
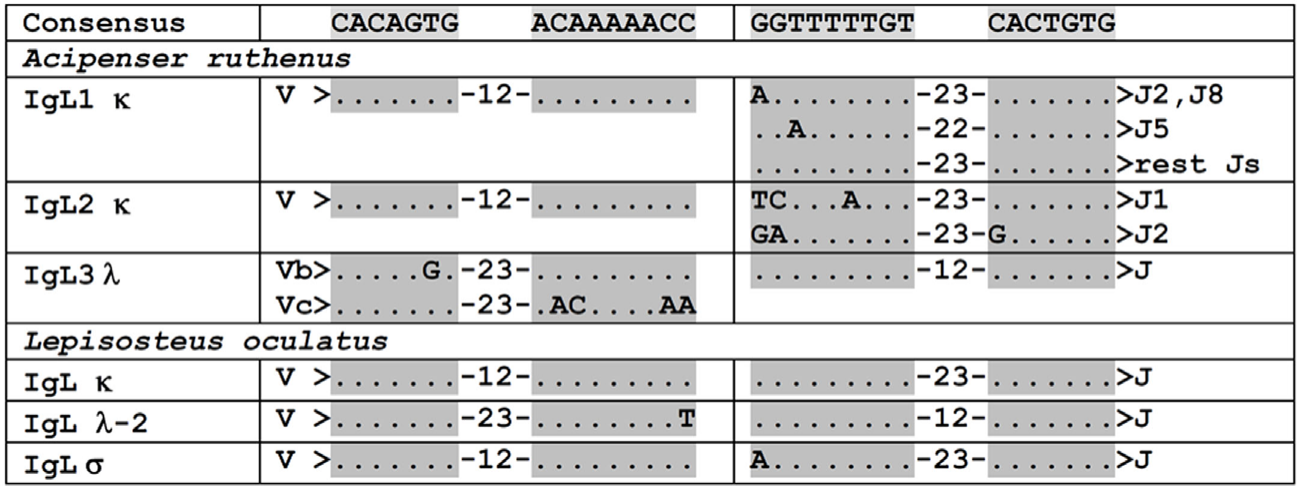
VL and J segments in sterlet (*Acipenser ruthenus*) and spotted gar (*Lepisosteus oculatus*) possess recombination signal sequences of both κ and λ types. Dots designate nucleotides matched with the consensus sequence shown above. The numbers within hyphens stand for spacer length in nucleotides between gray shaded heptamers and nanomers.

To better understand the organization of the sterlet IgL1 genes at the genomic level, we examined the genomic scaffolds of the D specimen. Two genome assemblies (B1 and B2) generated in the Institute of Molecular and Cellular Biology were used as a reference. Scaffold 16759 of the D genome was found to contain two V1.3 (one of which is a pseudogene), one V1.2, six J, and a single C2 gene segments. Scaffolds 43091 and 33312 contained a C1 gene and 3 or 4 J segments each (Figure 3). As expected, all identified V and J segments had functional RSS of the κ type (12/23, Figure 4). When searched at the level of cDNA, the scaffold-specific J segments were found only in association with their C gene neighbors (Figure S5 in Supplementary Material). The 3′-UTR sequences flanking the C1 and C2 genes showed only about 50% identical nucleotides (Figure 3, denoted by asterisks). In contrast, C1a- (the B genomes), C1b-, and C1d-containing (the D genome) scaffolds were similar to each other by 93−97% in the overlapping regions.

**Figure 5:**
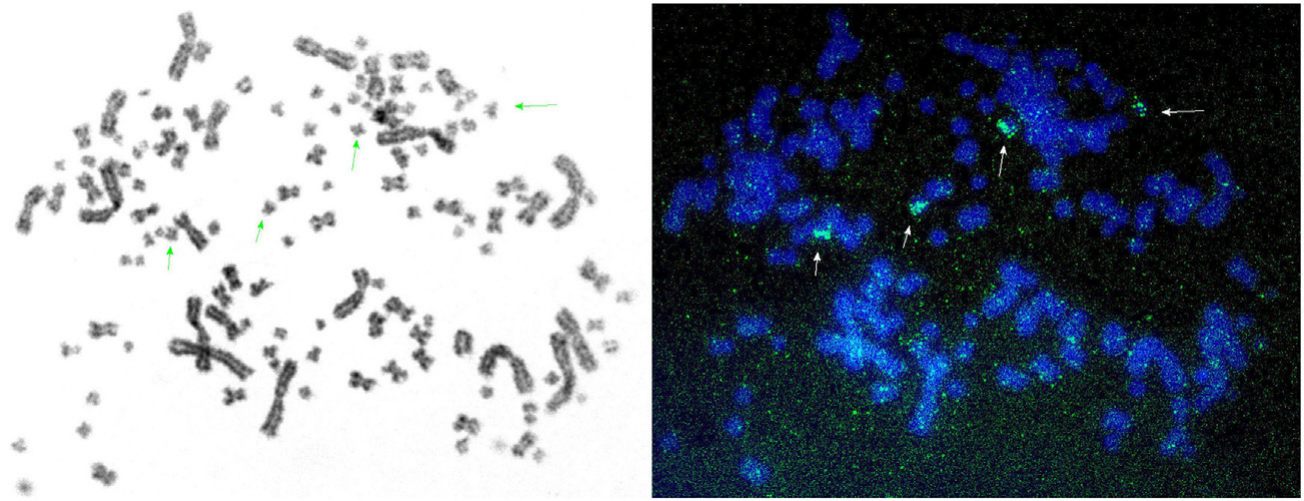
Fluorescence *in situ* hybridization shows that sterlet possesses two IgL1 κ loci: IgL1A and IgL1B. The figure represents a series of images of sterlet chromosomes hybridized with IgL1 V1.1-specific probe. Specific fluorescence signals are marked with arrows. Adjusting of brightness and contrast was performed without excluding background fluorescence.

All these findings showed that there are two distinct IgL1 loci in sterlet (Table 1). To further examine if these loci resulted from segmental or chromosomal duplications, we performed FISH hybridization using the V1.1-specific probe. Four hybridization signals were detected on small sterlet chromosomes (Figure 5). Therefore, we conclude that IgL1A and IgL1B loci are located on different chromosomes in sterlet. Previously, Lundquist et al. (27) have demonstrated by Southern blotting that the Siberian sturgeon genome has a larger number of IgL1 CL genes than the sterlet. Consistent with that finding, the results of our analysis of the sturgeon transcriptomes showed the presence of additional IgL1 C gene variants (not shown). Most probably this is explained by additional chromosome duplication as both the Siberian and Chinese sturgeons are known to have a higher ploidy level than sterlet with about twice as many chromosomes and double genome size (43).

#### V-J Recombination

Of 10 identified germline J segments, 9 have GT dinucleotide at the RSS-proximal end (Figure S5A in Supplementary Material). The V gene segments also showed conservation at the RSS-proximal ends. All the studied V1.1 genes contained the GTGTTCA sequence followed by RSS. In the case of V1.2 and V1.3, the RSS was always preceded by the (C/T)CCTCTCA sequence. An analysis of the V-J junctions in the cloned IgL1 cDNAs showed the absence of randomly added nucleotides, suggesting that terminal deoxynucleotidyl transferase (TdT) is not expressed at the time of IgL1 rearrangement. All V1.1.-J junctions and most of the V1.2/1.3-J junctions contained nucleotides derived from either V or J segments only (Figures S5B,C in Supplementary Material). In some of the V1.2/1.3-J junctions we found P-nucleotides. The presence of the GT dinucleotide resulted in an invariable valine residue in the V1.1-J junctions. Most of the V1.2/1.3-J junctions contained a proline residue encoded by CCN codons (Figure S5C in Supplementary Material). Based on this analysis, it can be concluded that the CDR3 diversity in the IgL1A and IgL1B chains is mainly determined by the inherited V gene repertoire.

### IgL2

#### Diversity of VLs and CLs

**Figure 6:**
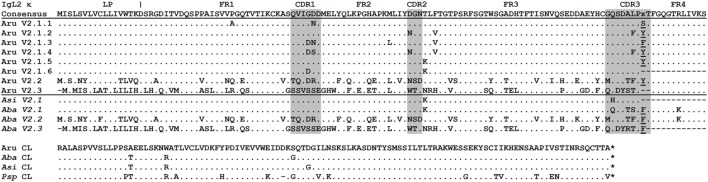
Sterlet IgL2 κ immunoglobulin light chains have limited VL repertoire. Sterlet (Aru) cloned sequences (MG029338–40, 43–45, 47) are shown together with American paddlefish (Psp), Siberian, and Chinese sturgeon (Aba and Asi) transcriptomic sequences (nucleotide sequences are provided in the Figure S7 in Supplementary Material). The FR and CDR regions predicted according to the IMGT standard (42) are shaded in gray. Dots represent identical to consensus amino acid residues, dashes—gaps introduced for alignment, asterisks— stop codons. Leader peptide (LP) boundaries are shown according to exon–intron structure in the genomic DNA. Amino acid residues at the V-J junction are underlined.

In total, we cloned 28 IgL2 cDNAs, with 13 of them being unique. The IgL2 CL domain was 46–49% identical to IgL1 C1 and C2 and showed homology to IgL κ chains in the BLASTP search against protein databases. The V genes of the cloned IgL2 cDNAs were subdivided into a V2.1 family containing five (V2.1.1–V2.1.5) closely related (95%) genes and two unique genes, designated V2.2 and V2.3 (Figure 6). One more variant, V2.1.6, representing about 7.8% of all IgL2 cDNAs was found in the Miseq dataset (Table S4 in Supplementary Material). All the cDNAs contained the same CL gene and one of two possible J segments (J1 or J2). A comparison of the IgL1 and IgL2 V regions showed 38–60% sequence identity at the amino acid level.

#### Structure of the IgL2 Locus

The search in the D genome revealed that scaffold 30517 contained V2.1.5, V2.2, V2.3, J1, J2, and C segments (Figure 3). The compactness of this locus is noteworthy (only 9,600 nt between V2.1.5 and C segments). The scaffold terminates near V2.1.5, and we believe that other V2.1 variants may be encoded further upstream. The identified V2.1 sequences differ from each other by a few amino acid residues located in the CDR1-3 regions (Figure 6). In contrast to the V1.1 and V1.2, the V2 domains did not differ in the length of CDRs. The only exception was V2.3 with CDR3 shortened by two residues. The presence of numerous identical cDNAs for V2.1.1–2.1.6 in the Miseq dataset and among the cloned cDNAs indicates that their CDR diversity is inherited, rather than resulting from somatic hypermutation. Therefore, the sterlet IgL2 locus has a typical translocon organization: it contains at least six V2.1, one V2.2, one V2.3, two J, and one C gene segment (Table 1). The RSS of the IgL2 gene segments belong to the κ-type (12/23, Figure 4).

#### V-J Recombination

Only one of the 13 unique IgL2 cDNAs contained the J2 segment, and all others had J1. An analysis of the MiSeq dataset similarly revealed that the J2 segment is used only in ~10% of the rearranged sequences. The bias in the use of two J-segments may be explained by a larger distance of J2 from the V segments or by differences in the RSS of J1 and J2 (Figure 4). A further search in the MiSeq dataset uncovered only three V2.1-J junction variants in the majority of IgL2 cDNAs. 75.8% of cDNAs encoded tyrosine or tryptophan depending on the use of J1 or J2, 11.2% encoded phenylalanine, and 5.7% contained a deletion of two codons (PY/WTFGQG/PFTFGQG/PFGQG) (Figure S6 in Supplementary Material).

### IgL3

**Figure 7:**
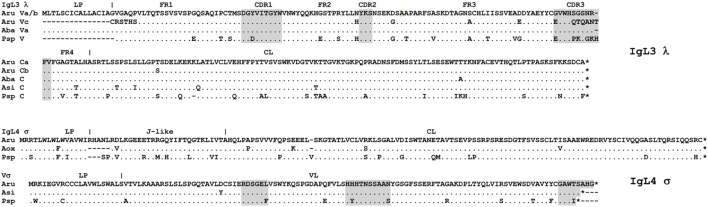
Sterlet IgL3 λ and IgL4 σ immunoglobulin light chains are homogenous. Sterlet (Aru) genomic (IgL3 Vc and IgL4 V-σ), and cloned sequences (IgL3 a/b, MG029351/MG029352 and IgL4, MG029353) are shown with American paddlefish (Psp), Siberian, Chinese, and Atlantic sturgeon (Aba, Asi, and Aox) transcriptomic sequences (nucleotide sequences are provided in the Figure S7 in Supplementary Material). FR and CDR regions predicted according to the IMGT standard (42) are shaded by gray. Dots represent identical to consensus amino acid residues, dashes—gaps introduced for alignment, asterisks—stop codons. Leader peptide (LP) boundaries are shown according to the exon–intron structure in the genomic DNA.

IgL3 identification was of particular interest as this isotype showed a high similarity to IgL λ chains of cartilaginous fish and tetrapods (up to 54 and 49% identical residues in CLs and VLs, respectively) but poorly (<40%) matched the teleost IgL “λ” chains. We found only two IgL3 variants in the sterlet transcriptome and in the MiSeq dataset. Designated IgL3a and IgL3b, these variants differed by five nucleotide substitutions, of which only one in the C region was non-synonymous (Figure 7). Both variants were cloned. Given an approximately equal proportion of IgL3a and IgL3b among the cloned cDNAs and MiSeq generated sequences, we consider them to be allelic variants. A search in the genomic sequences revealed two relevant scaffolds (Figure 3). The Vb gene was found in the scaffold 23972 and the J-Ca pair in the scaffold 16338. The latter scaffold also contains the gene for intraflagellar transport protein 81 (*IFT81*). Upstream of the Vb, there is a potential V pseudogene (Vc) that lacks an exon for LP and has disrupted RSS (Figures 3 and 4). The Vb and J segments were found to have λ type organization of RSS (23/12, Figure 4). Therefore, we conclude that sterlet has a single IgL3 cluster (V-J-C) with RSS of the λ-type (Table 1). According to the MiSeq dataset, there is no V-J junctional variability in the IgL3 transcripts (Figure S6 in Supplementary Material). Therefore, the IgL3 locus encodes light chains with the homogeneous V regions. An analysis of the sturgeon and paddlefish transcriptomic data revealed a single closely related IgL3 sequence in each of these species (Figure 7).

### IgL4

The sterlet spleen transcriptome contained a few cDNAs encoding an unusual IgL-like protein with a leader peptide, a diverged J-like region, and a CL domain. The latter was 52% identical to the Cσ of nurse shark. The dataset of the 5′-RACE PCR fragments obtained with the C region-specific primers showed the same sequence devoid of any V regions. In the D genome assembly, we found a scaffold (23288) containing all exons for the IgL4 polypeptide. The sequence analysis showed the presence of typical AG/GT splice sites flanking the LP, J, and C exons. No V-region genes were found between the exons for LP and J-segment (Figure 3). The J-segment lacked functional RSS. Nevertheless, a further search revealed the presence of a σ-like V gene segment in the sterlet genomic and transcriptomic sequences (Figure 7). The gene has a neighboring LP-coding exon but lacks typical RSS. Therefore, sterlet possesses two IgL σ-like genes, of which one encodes a shortened VL domain and another one a CL domain with an elongated J-like sequence at the N-terminus. Both polypeptides are presumably secreted as their LPs are cleavable according to the SignalP analysis. The CL domain retained a cysteine residue at the N-terminus, which is typically responsible for disulfide bonding with IgH chains.

The absence of transcripts for conventional VJC chains could be interpreted as evidence of the IgL4 locus aberrancy. However, we found similar transcripts for both VL and JCL parts of IgL4 in all studied Acipenseriformes, including American paddlefish. The VL and JCL polypeptides of sterlet and paddlefish share 90 and 83% identical residues, respectively (Figure 7). This strong conservation in species that diverged roughly 185 MYR ago (44) suggests that the locus may be functional.

### Sterlet IgL Expression

**Figure 8:**
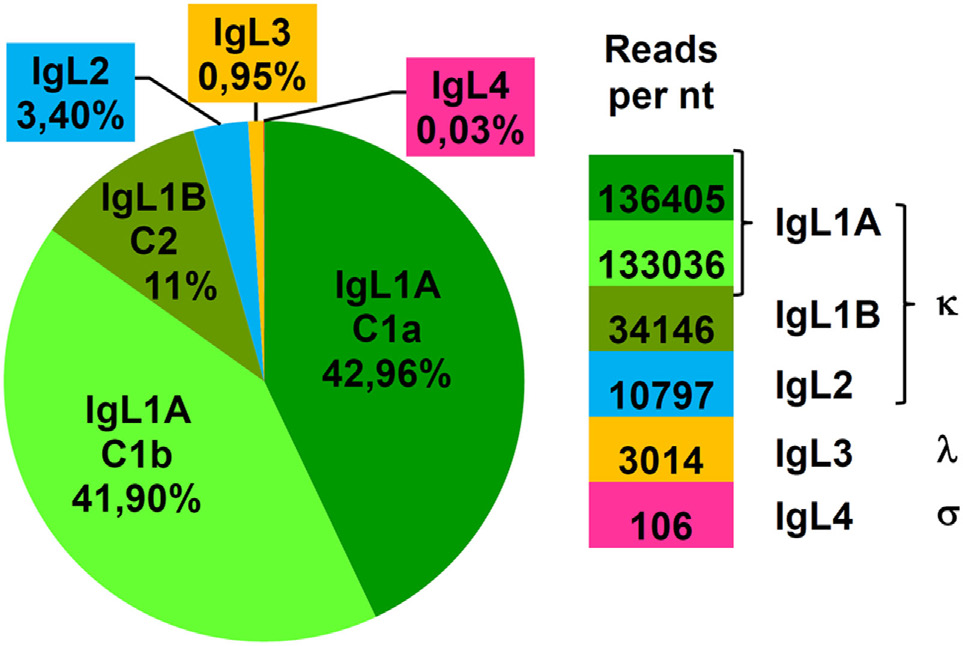
IgL κ loci make a major contribution to the expressed IgL repertoire of sterlet. The expression level of different IgL loci was estimated by counting reads in B1 spleen transcriptome matching the corresponding C segment (IgL1A/1B, IgL2-4). The sensibility of the algorithm used was sufficient to discriminate diverged C1a and C1b (IgL1A) but not similar C2c and C2d (IgL1B) segments. The mean number of reads per 1 nucleotide of corresponding C segment is shown at the right.

To estimate the expression level of the sterlet IgL chains in the spleen, we used the Genomecov program (40) that calculates the number of transcriptome reads per unit length of the CL genes. The results showed that IgL1A, IgL1B, IgL2, and IgL3 accounted for 85, 11, 3, and 1% of the sterlet spleen CL transcripts, correspondingly (Figure 8). Expression of the non-rearrangeable IgL4 locus was the lowest with −0.03%. The expression levels of the IgL isotypes roughly correlate with the number of the V genes found at each locus, and this is consistent with the stochastic model of IgL expression regulation (45).

### IgL Genes of Polypteriformes and Holostei

**Figure 9:**
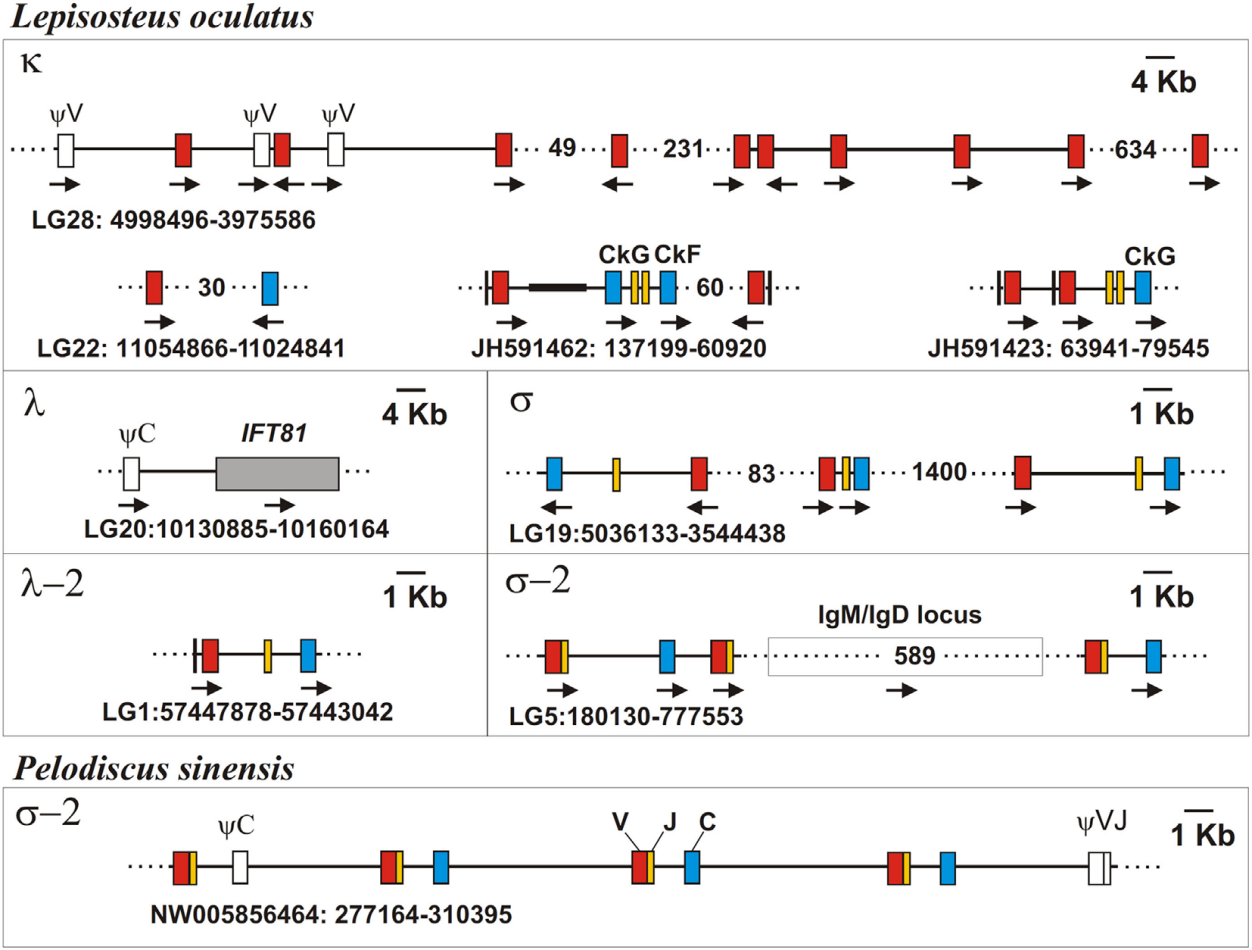
Spotted gar (*Lepisosteus oculatus*) genome contains IgL genes of five isotypes. Chinese softshell turtle (*Pelodiscus sinensis*) genome contains σ-2 IgL loci, as well. The exons encoding leader peptides (L) are denoted by vertical lines, variable (V), joining (J), and constant (C) segments are denoted by red, yellow, and blue rectangles, respectively. Pseudo genes are denoted by white rectangles. Black bold line on scaffolds marks NNN-stretch area on the JH591462 contig. The transcriptional direction of all genomic elements is showed by arrows. Gaps between distant scaffold regions are denoted by dotted lines and the distances between them are shown in Kbs.

The recent sequencing of the spotted gar (*L. oculatus*) genome and transcriptomes provided a possibility to use bioinformatics approaches for the identification and primary characterization of the IgL genes in this holostean species. The current version of the spotted gar genome (LepOcu1) was found to contain IgL genes in scaffolds mapped to six chromosomes (linkage groups) and in a group of unplaced scaffolds (Figure 9). Linkage group 1 contained a VJC cluster with RSS organized in a λ-like way. The encoded IgL chain showed the highest similarity to the teleost “λ” chains. Linkage group 5 contained two genes encoding σ-2-like IgLs. Both of these genes had V and J segments joined at the genomic level. Interestingly, the genes were found to flank the IgH locus (Figure 9). In the annotation to the current version of the gar genome, these genes are erroneously designated as κ-like. The linkage group 19 contains three IgL V-J-C clusters encoding σ-like chains. Next, we found about 150 structurally related κ-like VL genes on chromosome 28 and numerous unplaced genomic fragments (Figure 9). Of these, 107 appear to be functional as they do not contain stop codons or frame-shift mutations. Just a few of the latter scaffolds contained JL and CL gene segments suggesting thereby the typical translocon organization of the κ genes in gar. A potential κ-like pseudogene consisting of a Vκ and a Cκ gene segment was revealed in the linkage group 22. All the genes except those on chromosome 1 showed κ-characteristic RSS organization (Figure 4). Finally, we found a CL pseudogene containing a frame-shift mutation in the linkage group 20. At the nucleotide level, the pseudogene was 60% identical to the sterlet IgL3 CL and, like the IgL3 locus, was closely linked to the *IFT81* gene (Figures 3 and 9). Seven transcripts representing the rearranged diversity of gar IgLs were extracted from the TSA database for subsequent phylogenetic analysis. This set was extended by the sequence of the predicted transcript of the IgL pseudogene from the linkage group 22. We also used the sequence of the gar Cλ pseudogene from linkage group 20 in the analysis of CL gene relationships. The frame-shifting mutation in this sequence was corrected to provide proper alignment at the amino acid level.

The IgL sequences of Polypteriformes were mined from the Fish T1K database.^5^ This resource contains RNAseq data for a thousand of ray-finned fish species including, among others, two polypterids—saddled bichir (*P. endlicheri*) and ropefish (*E. calabaricus*). The bichir mRNAs have been obtained from gills and those of ropefish—from a mixture of organs (liver, gill, brain, gonads). The search revealed only a moderate diversity of expressed IgL chains in these tissues. Five of the most diverged IgL VJC sequences were chosen for further analysis (Figure S7 in Supplementary Material). It is clear, however, that the IgL repertoire in polypterids is more diverse. The database contains a few partial transcripts for CL domain fragments that showed weak (29–47% identical residues) similarity to the chosen polypterid and known IgL chains. Further experimental studies would be necessary to characterize polypterid IgL diversity in more detail.

### Phylogenetic Analyses

**Figure 10:**
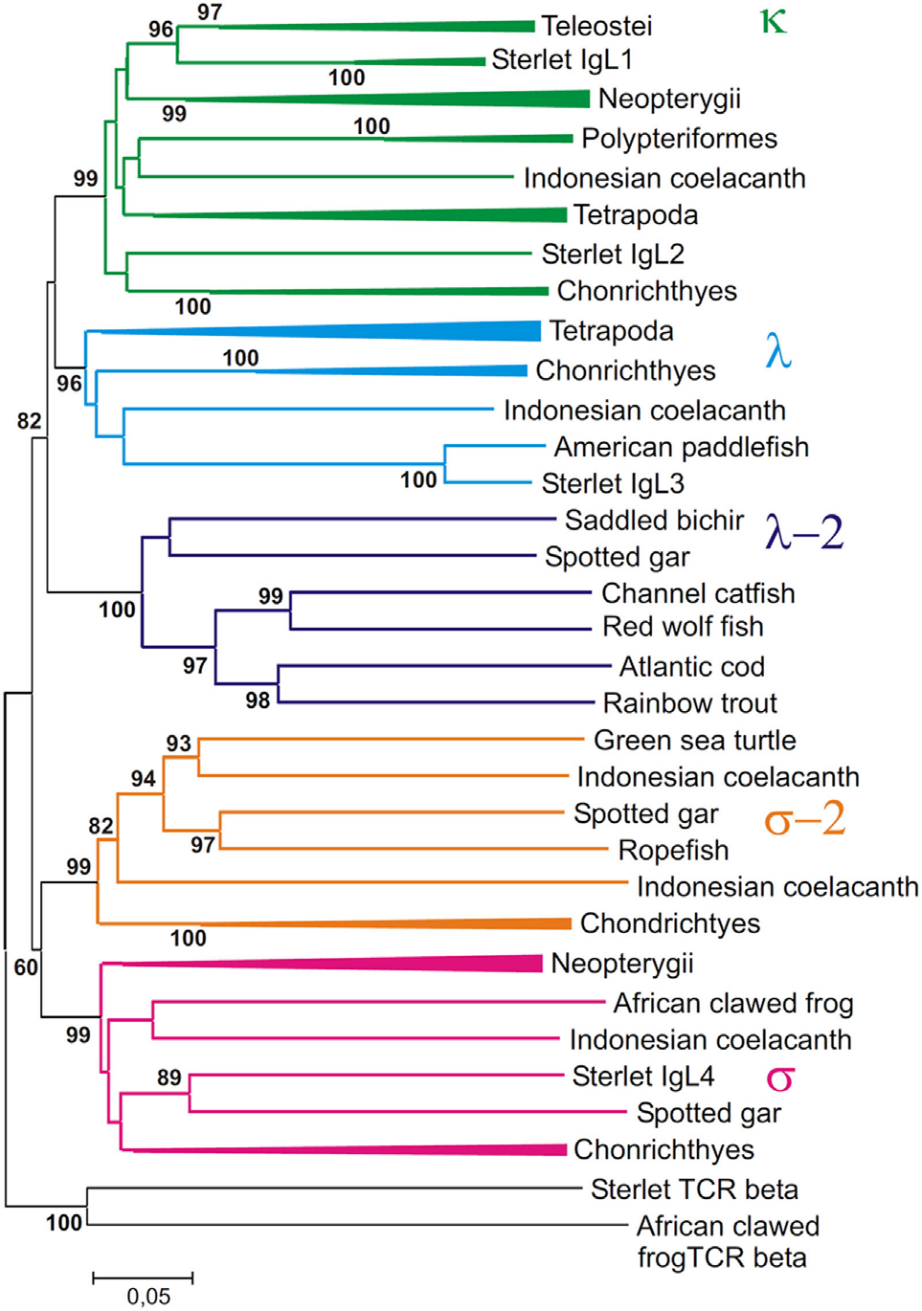
Phylogenetic analysis of full length IgL sequences (VJC) shows that they form five clades. The tree was constructed by the Neighbor-joining (NJ) method using nucleotide sequences after amino acid alignment. The bootstrap test (500 replicates) values equal or higher than 70% are only shown. Maximum likelihood and minimum evolution trees were essentially the same as the NJ tree in the major branching patterns. For the better compactness, some clades were compressed. A detailed tree may be found at the Figure S8 in Supplementary Material.

**Figure 11:**
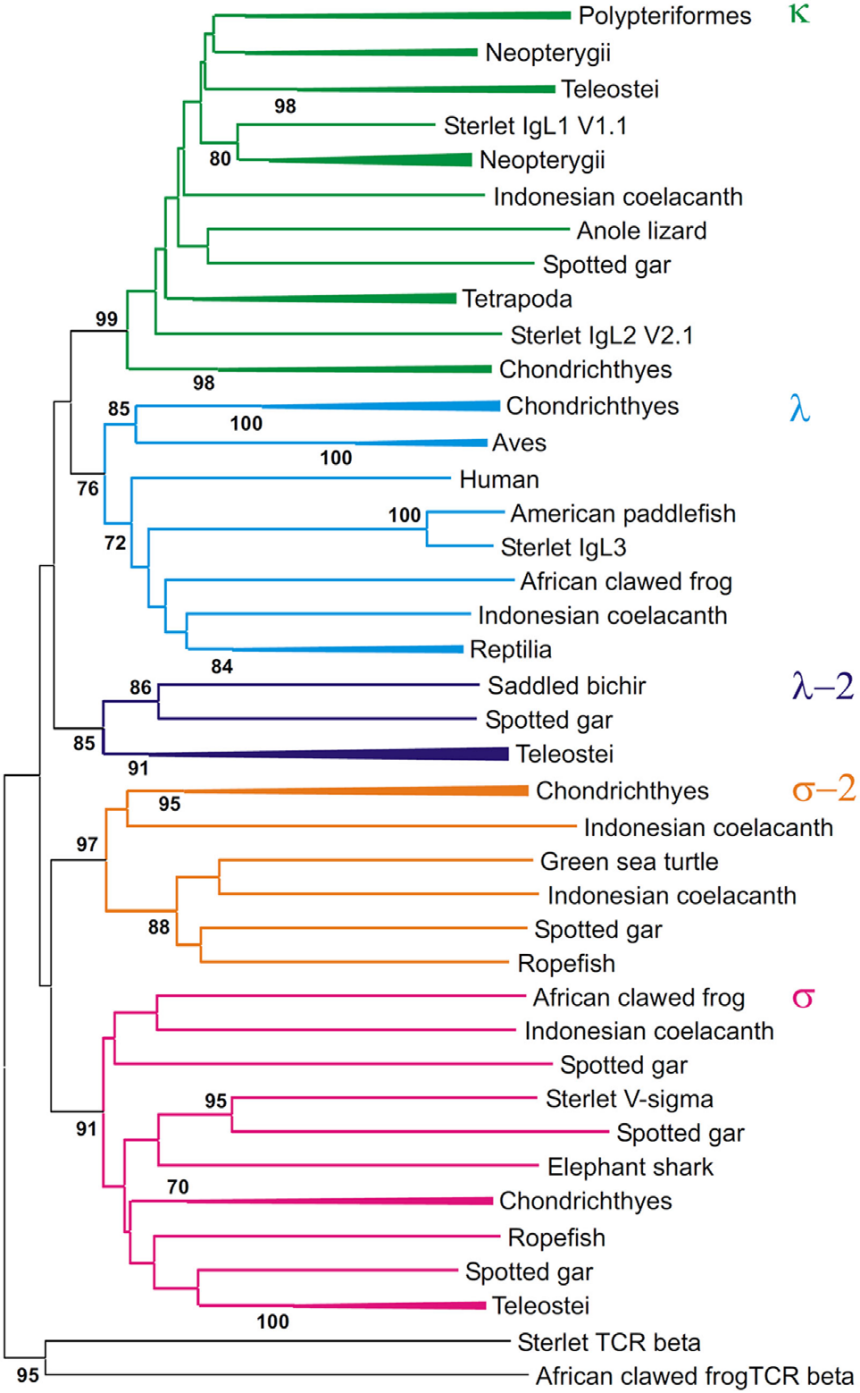
Phylogenetic analysis of VL sequences shows that they form five clades. The tree was constructed by the Neighbor-joining (NJ) method using nucleotide sequences after amino acid alignment. The bootstrap test (500 replicates) values equal or higher than 70% are only shown. Maximum likelihood and minimum evolution trees were essentially the same as the NJ tree in the major branching patterns. For the better compactness, some clades were compressed. A detailed tree may be found at the Figure S9 in Supplementary Material.

**Figure 12:**
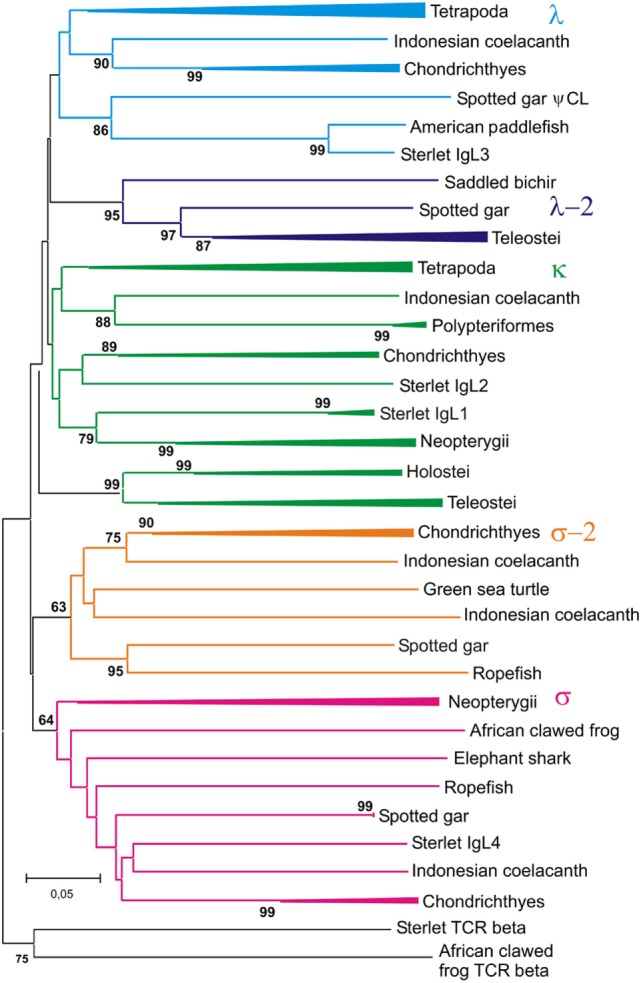
Phylogenetic analysis of CL sequences shows that Cλ and Cλ-2 sequences form two different clades. The tree was constructed by the Neighbor-joining (NJ) method using nucleotide sequences after amino acid alignment. The bootstrap test (500 replicates) values equal or higher than 70% are only shown. Maximum likelihood and minimum evolution trees were essentially the same as the NJ tree in the major branching patterns. For the better compactness, some clades were compressed. A detailed tree may be found at the Figure S10 in Supplementary Material.

The dataset of IgL sequences gathered in this study was, to our knowledge, the first representing all the main lineages of fish. The VJC, V, and C sequences from this dataset were used to generate a series of phylogenetic trees using MEGA6 software. Various alignments and tree generation methods (NJ, ML, and ME) were tested. The representative NJ trees are shown in Figures 10–12.

First of all, the analysis of the VJC and V sequences showed the statistically supported subdivision of IgLs into five major groups (Figures 10 and 11). Four of the groups corresponded to the previously described κ, λ, σ, and σ-2 isotypes (16). The fifth included teleostean IgLs that are currently thought to be orthologs to λ chains. However, in the trees generated the cluster of teleostean λ chains showed sister-group relationships to both the κ and λ chain clusters. The subdivision of VJC and V sequences into five groups was highly stable and tolerant to variations in sequence alignment and tree generation settings.

The tree resolution allowed the unequivocal definition of isotypes of the newly identified ray-finned fish IgL sequences. Acipenseriformes IgL1A, IgL1B, and IgL2 as well as representatives of the major group of the gar IgL genes from chromosomes 22, 28, and unplaced genomic regions (Figure 9), clustered together with κ chains. The κ chain cluster included also two of five chosen polypterid IgL sequences. Sterlet IgL4, one of the polypterid IgLs and sequences of three gar IgL genes from chromosome 19 clustered with σ chains. The gar IgL sequence encoded by the gene from chromosome 1 and one of the polypterid IgLs were found to cluster with teleostean “λ” chains. Most interestingly, none of the Acipenseriformes IgL sequences fell into the latter group. Instead, sterlet IgL3 and its paddlefish counterpart clustered with “true” λ chains. Based on these results we finally designated sterlet IgL chains as κ1A, κ1B, κ2, λ, and σ (Tables 1 and 2).

**Table 2:** Vertebrate IgL isotypes and their synonyms.

Taxon	Species	κ	λ	σ	σ-2	λ-2	Reference
Cartilaginous fish	Nurse shark	NS4	NS3	Type IV/σ	NS5/σ-cart		(16, 46)
						
	Horned shark	Type III	Type II	Type IV/σ	Type I/σ-cart		
						
	Little skate	ND	Type II	Type IV/σ	Type I/σ-cart		
							
	Elephant shark	κ	λ	σ	σ′/σ-prime		(47)
							
Lobe-finned fish	African coelacanth	κ	λ	σ	σ-2		(48)
							
	Indonesian coelacanth	κ	λ	σ	σ-2		
							
Amphibians	African clawed frog	L1/ρ	Type III	L2/σ			(6–8)
							
Reptiles	Sea green turtle	κ	λ		σ-2		
							
	Chinese softshell turtle	κ	λ		σ-2		
							
	Painted turtle	κ	λ		σ-2		
							
	Anole lizard	κ	λ				(49)
							
	Asian glass lizard		λ				(5)
							
	King cobra		λ				(5)
							
	Chinese alligator	κ	λ				(50)
							
Mammals		κ	λ				
							
Birds			λ				(51)
							
Polypters	Ropefish	κ	ND	σ	σ-2	ND	
							
	Saddled bichir	κ	ND	σ	ND	λ-2	
							
Acipenseriforms	Sterlet	κ1A, κ1B, κ2	λ	σ			
							
	American paddlefish	κ	λ	σ			
							
Holosteans	Spotted gar	κ	ψλ	σ	σ-2	λ-2	
							
Teleosts	Rainbow trout	Type 1, type 3/L1, L3/κF, κG		Type 2/L2		λ	(18)
							
	Catfish	Type 1, type 3/F, G		Type 2/L2		λ	(11, 17, 52, 53)
						
	Atlantic cod	Type 1, type 3/L1, L3		Type 2/L2		λ	
							
	Zebrafish	Type 1, type 3/L1, L3		Type 2/L2			(17, 52, 53)

							
	Common carp	Type 1, type 3/L1A, L1B, L3		Type 2/L2		

*Isotypes identified in this study is shown by green (genome data), blue (transcriptome data), or red (both) color*.

*ND, not determined*.

The trees generated show that teleostean “λ” chains represent, in fact, a distinct isotype that is paralogs to λ chains. We designated this isotype λ-2 to take into account its λ-characteristic RSS organization at the genomic level. It is obvious that a common ancestor of ray-finned fish possessed both λ and λ-2 IgLs. Indeed, the results of the phylogenetic analysis of the CL sequences demonstrated that the CL pseudogene found on gar chromosome 20 is ortholog to the CL of Acipenseriformes IgL3 (Figure 12). Thus, we conclude that, apart from the functional λ-2 gene, gar has a pseudogene for the “true” λ chains. It may be suggested that the latter have been lost in the Neopterygii lineage before the radiation of Holostei and Teleostei.

Relationships of the CL sequences are known to be less obvious than those of VL. Like in the previous studies (16, 54), our analysis showed mixed branching of Cκ and Cλ sequences (Figure 12). The reasons for poor resolution of the Cκ and Cλ relationships are not clear. The most probable explanation is that sequence exchange (gene conversion or exon shuffling) might have happened in these two loci at some stage of evolution. Further accumulation of genomic sequences from cartilaginous and polypterid fish would be necessary to understand these events better.

In contrast to Cκ and Cλ sequences, the Cσ, Cσ-2, and Cλ-2 formed stable clusters with 64–99% bootstrap support. Furthermore, the results of CL analysis shed light on the evolution of teleostean IgL κ chains. The latter are known to consist of two subtypes, L1/κG and L3/κF, which strongly differ by their C regions but share similar VL domains [reviewed in Ref. (4, 52)]. There are two variants of Cκ regions in spotted gar as well. One of these is clustered with teleostean L1/κGs and the other with teleostean L3/κFs (Figure S10 in Supplementary Material). 99% bootstrap support clearly indicates that this particular Cκ duplication had occurred in the Neopterygii lineage before the radiation of holosteans and teleosts. Interestingly, the genes for CκG and CκF are closely linked in the gar genome (Figure 9). This fact suggests their origin by segmental duplication and explains a surprising association of strongly diverged CκG and CκF with structurally similar V region genes in the teleost fish. Duplications of Cκ loci in Acipenseriformes most probably have occurred independently. Although Acipenseriformes Cκ1 regions tend to cluster with teleostean L1/κG subtypes, the Cκ2 region is closer to Cκ of cartilaginous fish.

**Figure 13:**
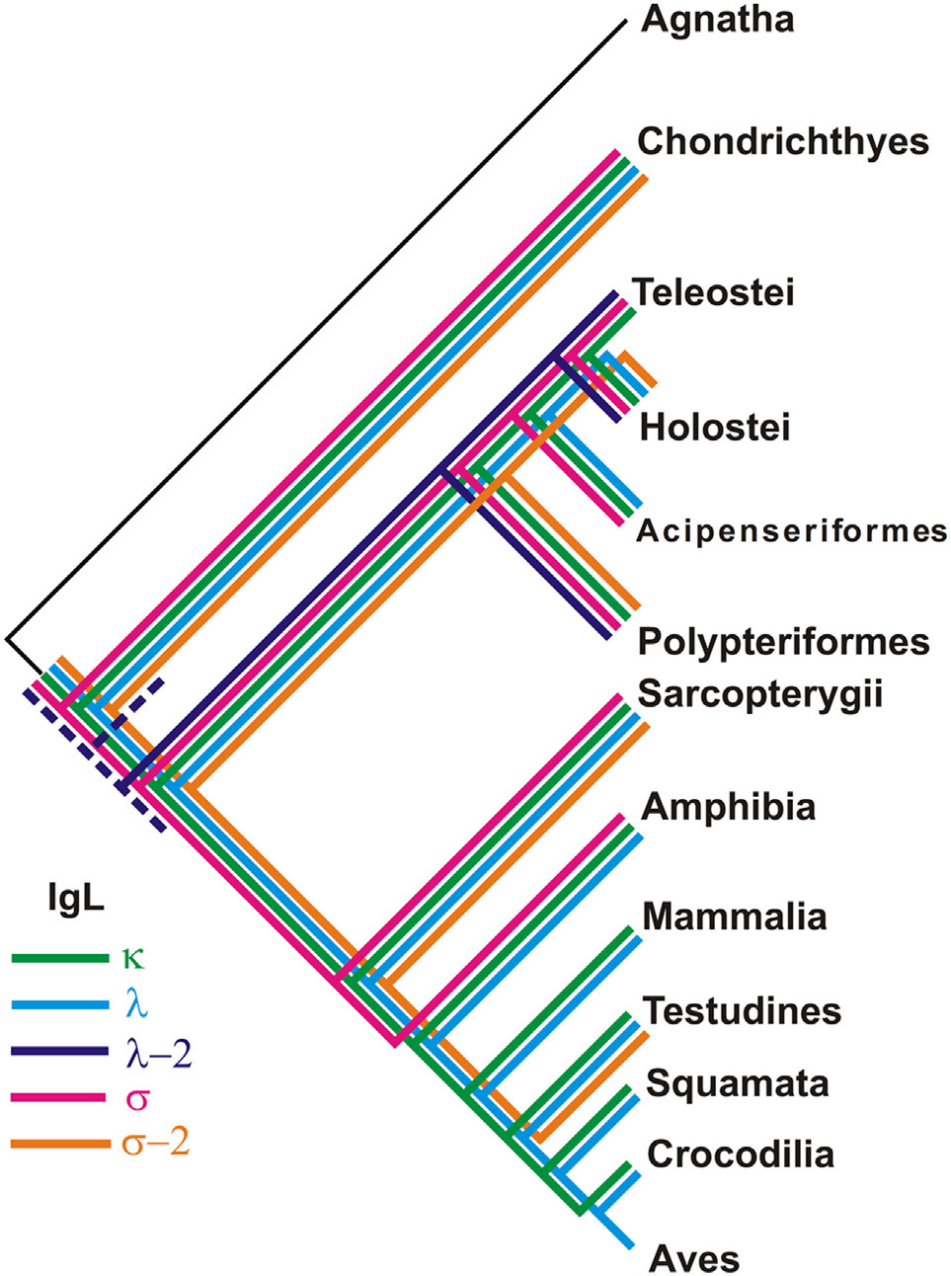
Different taxonomic groups of jawed vertebrates retained unique sets of IgL isotypes.

One more phylogenetic finding worth mentioning is that the σ-2 isotype is spread broader than it was previously recognized. Originally defined as cartilaginous fish-specific, this isotype has been recently revealed in coelacanth (48). Our data show that σ-2 has also been retained by polypterid and holostean fish. Most strikingly, we also found σ-2 genes in turtles (Table 2; Figures 11–13). The current version of the spotted gar genome contains two σ-2 loci, while that of Chinese softshell turtle (*Pelodiscus sinensis*) has four (Figure 9). Similar to coelacanth, the σ-2 V and J segments are joined in the gar and turtle genomes.

The description of the elephant shark genome reported the presence of IgL κ and λ, but no IgL σ chains in this species (47). The σ-2 isotype was represented by a single C region gene. Our analysis of the recently published elephant shark transcriptomes revealed both IgL σ and σ-2 chains in this species (Figures S9 and S10 in Supplementary Material). Thereby, we conclude that chimeras are similar to sharks in retaining four IgL isotypes (Table 2).

## Discussion

In this report, we described the sequences and evolution of IgL chains in representatives of three lineages of non-teleostean ray-finned fish: Acipenseriformes, Holostei, and Polypteriformes. Using both *in silico* and experimental searches, we showed the presence of three IgL isotypes in Acipenseriformes, four in holosteans and at least four in polypterids. These data fill an important gap among teleostean, cartilaginous, and lobe-finned fish. Specifically, we found that the counterparts of IgL κ chains previously identified in the Siberian sturgeon (27, 28) are encoded by two distinct and highly related loci (κ1A and κ1B) in sterlet. The loci are localized on different chromosomes. Both comprise a single Cκ gene, a group of J segments and numerous VL gene segments. We found one more κ-like locus (κ2) in sterlet that is smaller in size (only 8 V genes) but is also organized in a translocon manner. Furthermore, sterlet has been shown to possess two additional IgL loci, λ and σ, both containing a single VJC gene combination. The λ locus encodes a homogeneous λ light chain as its rearrangement does not generate junctional variability. The σ locus appears to be non-rearrangeable. It produces distinct transcripts for a Vσ domain and for a Cσ domain with J-like sequence at the N-terminus. Both the Vσ and Cσ products contain cleavable leader peptides.

According to our data, 99% IgL mRNA in the sterlet spleen encode κ chains. The λ transcripts comprise only 1%. The relatively poor expression and the absence of heterogeneity suggest that λ chains may play only a minor and highly specialized role. The least expression level, 0.03%, was found in the case of the σ locus. Although this locus does not encode a typical light chain, its strong conservation in Acipenseriformes suggests a functional significance. It cannot be excluded that polypeptides encoded by sterlet IgL σ genes may serve as components of secreted antibodies or B cell receptors in a manner similar to VpreB and λ5 subunits of the mammalian surrogate light chain (55, 56). If so, it would be an interesting example of convergent evolution of the Ig genes.

One of the ways of functional specialization of IgL chains may be their non-stochastic association with IgH chains. Such differential IgL–IgH association has been described in humans, frogs, and teleost fish (18, 57–59). Acipenseriformes have two IgH isotypes, IgM and IgD. IgH δ chains are expressed at a much lower level and use only one of five VH gene families found in association with μ chains (26). With this in mind, it would be of interest to find out if sterlet and sturgeon IgM and IgD differ in their ability to associate with the IgL isotypes.

Phylogenetic analysis of the extended IgL dataset provided new insights into the evolution of IgL chains. Our results unequivocally demonstrate the subdivision of vertebrate IgLs into five major isotypes. The IgL chains, known as teleostean IgL “λ” orthologs, actually represent a distinct isotype that we designated λ-2. Apart from teleosts, this isotype is present in holostean and polypterid fish suggesting its emergence before the radiation of ray-finned fish (390–420 MYR ago). The λ-2 genes are, however, absent in Acipenseriformes fish that instead retained genes for the “true” λ chains. That the λ and λ-2 chains are paralogs rather than orthologs is evident not only from the topology of the phylogenetic trees. We found that the spotted gar genome, apart from the transcribed λ-2 locus on chromosome 1, contains a defective Cλ gene on chromosome 20 (Figure 9). This finding suggests a scenario in which a common ancestor of ray-finned fish possessed all five IgL isotypes. During the subsequent evolution, σ-2 and λ-2 have been lost in Acipenseriformes, while Teleostei have lost σ-2 and λ chains. Some teleost species have also lost λ-2 (52). Further studies are needed to reveal if polypterid fish have IgL λ. To date, the spotted gar is the only species possessing genes (functional and non-functional) for all the five IgL isotypes (Figure 13; Table 2).

Despite an intense search, we did not find λ-2 orthologs in cartilaginous and lobe-finned fish. The absence may be explained by either the loss of the λ-2 genes in these lineages or by the isotype emergence in ray-finned fish. We favor the former explanation and suggest that a common ancestor of gnathostomes possessed λ-2 together with the other IgL isotypes. There are several reasons to think so. First, κ, λ, and λ-2 clusters show sister-group relationships in the IgL trees (Figures 10 and 11). Second, the loss of a particular IgL isotype or isotype combination was a usual event during vertebrate evolution (Figure 13; Table 2). Third, λ-2 chains appear to play a minor role in humoral immunity as they are encoded by just 1–2 genes in all species known to possess them. Finally, it cannot be excluded that λ-2 genes have been retained in some not yet investigated cartilaginous or lobe finned fish. The latter possibility is well illustrated by our unexpected finding of σ-2 chains in turtles.

The data obtained in our study support the previous observation that different IgL isotypes retain the pattern of CDR1 and CDR2 lengths over hundreds of millions of years (16). Vσ-2 CDR1 and CDR2s have the same lengths (8 and 7 residues, respectively) in all species possessing these chains (Table S5 in Supplementary Material). Vσ show the longest CDR2 (9–10 residues). Furthermore, Vλ-2 may be distinguished by short length of both CDR1 (5–7 residues) and CDR2 (2–3 residues). The characteristic feature of κ chains is short CDR2 (3 residues) and high variability of the length of CDR1 (6–12 residues). In λ chains, the length of both CDR1 (3–9 residues) and CDR2 (3–7 residues) is highly variable. In terms of gene usage, κ and λ chains appear to be evolutionary more “successful” than the three other isotypes (Figure 13; Table 2). κ chains are present in the vast majority of vertebrates and play a major role in many of them. λ chains are broadly distributed in the tetrapod lineage. Criscitiello and Flajnik (16) have suggested that the length of CDR1 and CDR2 may be responsible for functional distinctions of the IgL isotypes by affecting topology of antigen-binding sites when associated with IgH V domains. If this is the case, κ and λ chains with their range of CDR lengths may be functionally more flexible. Such flexibility may explain their preferential retention in the evolution of vertebrates.

## Ethics Statement

This study was conducted in accordance with the recommendations of the Animal Research Guidelines of the Ethics Committee on Animal and Human Research of the Institute of Molecular and Cellular Biology (Novosibirsk, Russia). All the protocols were approved by the Ethics Committee on Animal and Human Research of the Institute of Molecular and Cellular Biology (Novosibirsk, Russia).

## Author Contributions

AT and SG designed all experiments. MSt, JG, SW, MSc, VT, and WW provided the draft of the D genome (*Acipenser ruthenus*). SG, SK, AM, and VT analyzed Acipenseridae Miseq, genomic and transcriptomic datasets. KB, AN, LM, and NC cloned and sequenced sterlet IgL cDNAs, analyzed their structure. DA carried out FISH analysis. AT performed blast analysis of all publicly available transcriptomes and genomes and accomplished phylogenetic analysis. AT and SG wrote the manuscript. All the authors contributed to the manuscript revision, read it, and approved the submitted version.

## Conflict of Interest Statement

The authors declare that the research was conducted in the absence of any commercial or financial relationships that could be construed as a potential conflict of interest.

## Footnotes

^1^https://blast.ncbi.nlm.nih.gov.

^2^www.ensembl.org.

^3^https://db.cngb.org/fisht1k/.

^4^https://figshare.com/s/e3de3df0e366d07ff87a.

^5^https://db.cngb.org/fisht1k/.
