# Supplementary material for: Diversity of Immunoglobulin Light Chain Genes in Non-Teleost Ray-Finned Fish Uncovers IgL Subdivision into Five Ancient Isotypes
Source: Front Immunol. 2018 May 28;9:1079. doi: 10.3389/fimmu.2018.01079 (PMC5985310; doi:10.3389/fimmu.2018.01079)
Supplement: Supplementary file 1 [file table_1.PDF]

Supplementary table 1. Statistics of sterlet and sturgeon genomes and transcriptomes obtained in the Institute of Molecular and Cellular Biology (sections 2.3 and 2.10).

Species	Specimen	Sex	Type	Read length	Scaffold >500	scaffold N50	Read pairs	Transcripts	ORFs	Complete ORFs
Acipenser ruthenus	B1	Female	gDNA	100	770689	1557	216379439			
	B2	Male		100	687858	947	222530849			
	B1	Female	polyA -RNA	100			94135627	407220	290955	134004
Acipenser baerii	C	Female		150			33562211	357182		
