# Supplementary material for: Diversity of Immunoglobulin Light Chain Genes in Non-Teleost Ray-Finned Fish Uncovers IgL Subdivision into Five Ancient Isotypes
Source: Front Immunol. 2018 May 28;9:1079. doi: 10.3389/fimmu.2018.01079 (PMC5985310; doi:10.3389/fimmu.2018.01079)
Supplement: Supplementary file 2 [file table_2.PDF]

Supplementary table 2. Sequences of primers used in this study.

**Primer sequence**	**Dir**	**Primer location**	**Application**
5′-AAGCAGTGGTATCAACGCAGAGT-3′	F	Universal for RACE cDNA ends	5′- and 3′-RACE, Miseq
**IgL1**			
5′-ACCAGGCAGACCAAGGTGGCTT-3′	R	C1/C2	Amplification for Miseq
5′-GAGACGGAGGAAGGAGCAGTG-3′	R	C1/C2	Reamplification for Miseq
5′-CCCTTCATGTCAACAGAATCCAC-3′	F	V1.1 5′UTR	cDNA cloning
5′-TGTTCAGACCTTTCAGAGCTCCT-3′	F	V1.2 5′UTR	
5′-AGAGCTGCCCTTCATGTCAACAC-3′	F	V1.3 5′UTR	
5′-CTAATAGTGTGCACTCGCTCCTCT-3′	R	C1	
5′-ATAGTGTGCACTGGCTCCTGC-3′	R	C2	
5′-AAGCAGCGGGATTCTCTTGCC-3′	R	C1/2 3′UTR	
5′-CAGGAATCCAGTGGACAGTATAC-3′	F	V1_FR1	Fish probe amplification
5′-TGCATCTTCAGCCTGGAC-3′	R	V1_FR3	
**IgL2**			
5′-GACTTGCTGTTGAGAATCCCGTCT-3′	R	C	Amplification for Miseq
5′-AGGAGGTAGCAGACTGACAACC-3′	R	C	Reamplification for Miseq
5′-ACATTGTCACAAGAAGAGTCACCATG-3′	F	V2.1 5′UTR	cDNA cloning
5′-ACCAGTTTAATGAGAACAGGCACA-3′	F	V2.2 5′UTR	
5′-ATTGCCACATGAACATTAAGATGTCA-3′	F	V2.3_5′UTR	
5′-GACAATGGTTTGACTTCCCAGCTAG-3′	R	3′UTR	
**IgL3**			
5′-CCTTCCAAGACACGCTCACTGT-3′	R	C	Amplification for Miseq
5′-GTGAAGGTGAGGACAGAGTTCC-3′	R	C	Reamplification for Miseq
5′-CTGTTTCAGAGCATCTAACCCGC-3′	F	5′UTR	cDNA cloning
5′-GAGGCCCGAATCGACTCGT-3′	R	3′UTR	
5′-AAGCACCCCGAGATATCTTCTACAC-3′	F	Vc_3′RACE	3′-RACE
5′-TGGAAATGATTAAATGGCAGGC-3′	R	Vc_5′RACE	5′-RACE
**IgL4**			
5′-CTGGTCACTGCTGTCTCATTGG-3′	R	C	Amplification for Miseq
5′-TGACGGTGCAGGGAGCTGATGGGCTGTTA-3′	R	C	Reamplification for Miseq
5′-GGGAAGAGGGAAGCTGCTGG-3′	F	5′UTR	cDNA cloning, 3′-RACE
5′-AAGGGCACACAATGTGAAGCTG-3′	R	3′UTR	cDNA cloning, 5′-RACE
