# Supplementary material for: Diversity of Immunoglobulin Light Chain Genes in Non-Teleost Ray-Finned Fish Uncovers IgL Subdivision into Five Ancient Isotypes
Source: Front Immunol. 2018 May 28;9:1079. doi: 10.3389/fimmu.2018.01079 (PMC5985310; doi:10.3389/fimmu.2018.01079)
Supplement: Supplementary file 3 [file table_3.PDF]

Supplementary table 3. Number of cloned sterlet IgL1 cDNAs, coding for V1.1-1.3 and C1-2 domains. cDNA clone without C is marked with asterisk.

	C1		C2		Sum	
	all	unique	all	unique	all	unique
V1.1	28	28	4	4	32+1*	32+1*
V1.2	2	2	13	11	15	13
V1.3	-	-	13	8	13	8
Sum		30		23		53+1*
