# Supplementary material for: Diversity of Immunoglobulin Light Chain Genes in Non-Teleost Ray-Finned Fish Uncovers IgL Subdivision into Five Ancient Isotypes
Source: Front Immunol. 2018 May 28;9:1079. doi: 10.3389/fimmu.2018.01079 (PMC5985310; doi:10.3389/fimmu.2018.01079)
Supplement: Supplementary file 4 [file table_4.PDF]

Supplementary table 4. Presence (+) of IgL2 VL sequences in the cloned and Miseq sterlet cDNAs, in transcriptomes (Tra) and genomes (Gen) of sterlet (B1, B2, D specimens), transcriptomes of Siberian (Aba) and Chinese (Asi) sturgeons.

IgL2	Cloned #	Miseq #	Miseq %	B1 Tra Spleen	B1 Gen	B2 Gen	D Gen	Aba Tra Spleen	Asi Tra Spleen+
V2.1.1	1	65	5,4			+	+		
V2.1.2	3	107	8,9				+		
V2.1.3	1	246	20,5			+	+		
V2.1.4	1	445	31,1			+			
V2.1.5	1	222	18,5	+	+	+	+	+	+
V2.1.6		94	7,8						
V2.2	2	14	1,2	+	+	+	+	+	+
V2.3	4			+	+	+	+	+	
