# Supplementary material for: Diversity of Immunoglobulin Light Chain Genes in Non-Teleost Ray-Finned Fish Uncovers IgL Subdivision into Five Ancient Isotypes
Source: Front Immunol. 2018 May 28;9:1079. doi: 10.3389/fimmu.2018.01079 (PMC5985310; doi:10.3389/fimmu.2018.01079)
Supplement: Supplementary file 5 [file table_5.PDF]

Supplementary Table 5. CDR length variation in VLs used for the phylogenetic analysis (2.10 section, Figure 11). The CDR length was determined according to the IMGT standard (42).

Isotype	Seq#	CDR1	CDR2
κ	20	6-12	3
λ	12	3-9	3-7
λ-2	6	5-7	2-3
σ	12	4-7	9-10
σ-2	8	8	7
