# Supplementary figures and images for: Diversity of Immunoglobulin Light Chain Genes in Non-Teleost Ray-Finned Fish Uncovers IgL Subdivision into Five Ancient Isotypes
Source: Front Immunol. 2018 May 28;9:1079. doi: 10.3389/fimmu.2018.01079 (PMC5985310; doi:10.3389/fimmu.2018.01079)

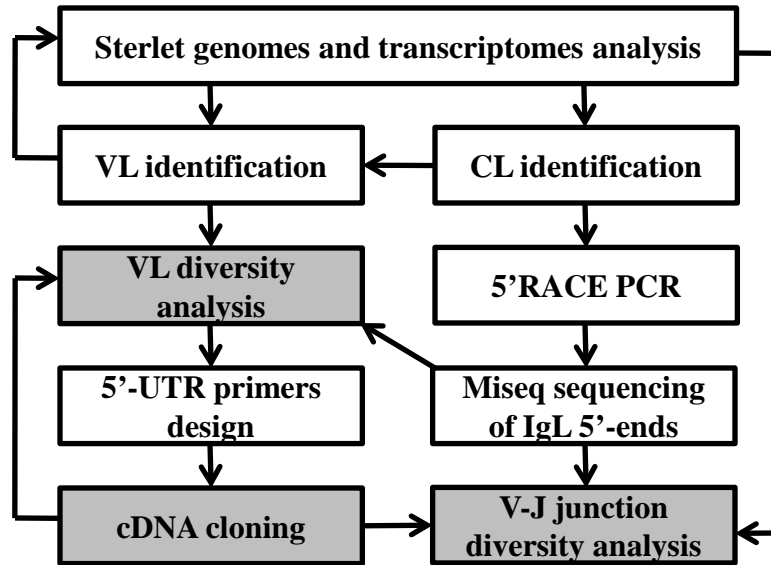

Supplementary figure 1. Strategy used for sterlet IgL repertoire characterization.

Supplement: Supplementary file 6 [file data_sheet_1.PDF]
