# Supplementary material for: Diversity of Immunoglobulin Light Chain Genes in Non-Teleost Ray-Finned Fish Uncovers IgL Subdivision into Five Ancient Isotypes
Source: Front Immunol. 2018 May 28;9:1079. doi: 10.3389/fimmu.2018.01079 (PMC5985310; doi:10.3389/fimmu.2018.01079)
Supplement: Supplementary file 7 [file data_sheet_2.PDF]

```
IgL1              LP         |           FR1                CDR1          FR2       CDR2                 FR3                    CDR3          FR4       Acc No
Consensus   MTFISIFIWALVICTQESSGQYTVTQTPAVKSVLPGDTVALSCKVSSAVYSNxxxxNYLAWYQQKPGEAPKLLIYxASTLQSGIPTRFSGSGSGTDFTLTISGVQAEDAGDYYCQSYHSP----SSGWVYTFGPGTKLVVK
      ArL1F  ......................................................TFSGSEQS.Y....E..........Y.IK.....................................H..ANVW------------------     AF130730
      ArL93  .....................V......T...............T.T...TASNY-H..T...............R..F.E.................................YL----N.KA.L........I..        1056
      ArL4E  .................................................QG.----.H.C.......GT......R.............C..............................L.I.----G.N..L........I..     AJ133187
      ArL2A  ..................G.........T....V.....T....T.....TYTNY-H..G......P........RT.Y....V.G..................................-------..Y.W..........          88
      ArL1B  .............................E...........N...N...LG.----T..H...............R....E...........................E.....V.V.YS----..RY.L........I..          89
      ArL2D  ...............................................P.DD.----YR.......S..V......W.............................E.......NS.Y.----..S.WL........V..     AJ236869
         01 -.........T.............L....R.......................DM----.R.................LT.N..........................V.........TA.Y-----...Y.............     MG029293
         02 -.............................L..G................HSDNYG-NR....K.....S......F.......................................F.Y.----..RG.............         294
         03 -..................................................----.H.................F.......................................F.Y.----G.SR.L........I..         295
         04 -...........L............I.........................----.R.................E.......................................E.YS----..-..............         296
         05 -.......................................T..........N.----KN.................A.......................................N.Y.SSREVF*--H...........         297
         06 -.............Y...................V................N.----I..................A..NL.............................AY.....L..-------.G.............         298
         07 -..................................................----.....F.............R...........................H...A....L.N.Y.N----.RE.............         299
         08 -.............................................RR....----DF.....K......T....W..TR.HWL......................I..A......A..-------.N.............         300
         09 -..L....................L..........................DSNG-.H......T..........Y..I..........................................-------.G.............         301
         10 -.................................................AN.----...................Y.........................................A.YS-----S.Y.L........I..         302
         11 -.......................................V..........V.----.H.................Y....................I.....................L.Y.----..R..FT.......D..  C1     303
         12 -.................................................LA.----S..................A..Y......................................A..-------.N.............         304
         13 -............................V.....................G.----DE.................T.............................E..........V.YS----..RY.............         305
         14 -.....................I......E...............T.T...TYNNY-QR.G...............NANI......................................V.N.----..RF.W..........         306
         15 -..............Y...................................----.H.................F.......................................F.Y.----G.SR.............         307
         16 -.......................................T.........H..----.W.................TT.N..........................V..V...........Y.----..RV.............         308
         17 -..................................................----...................R...........T.............R.....V.......E.N.----..S..W..........         309
         18 -..................................................N.----...................R...........................V..................-------.NL............         310
         19 -.........................................N..T.....NDGDD-D..................Y..R.E...................................Y.----..N..........-----         311
         20 -.....................I......G.....................----.R.T...............Y...........................V..................-------.NVF..........         312
         21 -.................................................HS.----.W.................R..N......................................L.DIGNIF..S..C...........         313
         22 -................................................A...----.R.................TT.I......................................A.Y.----..S..F..........         314
         23 -.........................................N.....V...TSYG-HR.................Y............................................-------.Y.L........I..         315
         24 -.......................................T............DSNG-HW.................L.......................................A.Y.----..S..............         316
         25 -.............................................N.....DSNG-.R....K.K...D......F..I.....................N.......S..........EP----...Y.............  C2     317
         26 -..................T...........................N.----.........T..........L.......................................F.Y.----..RD.............         318
         27 -..................................................----...................G..N......................................A..-------.G.F..........         319
         28 M.SLFLLVGTL.I.SA.V....I.M....SAL.T...ER.TIN..A..S.S.------.....L.T..........S.........A.............................QGYTS---------W...........  C1     320
         29 M.SLFLLVGTL.I.FA.V....I.M....SAL.A...ER.TIN..A..S.S.------.....L.T..........S.........A.............................Q.Y.SP--------W...........         321
         30 M.SLFLLVGTL.I.SA.V....I.M....SAL.AP..ER.TIN..A..S.S.------.....L.T..........S.........D.............................QGY.S---------............         322
         31 M.SLFLLVGTL.I.FA.V....I.M....SAL.A...ES.TIN..A..S.S.------.....L.T..........A.KS......A.............................QGY.S---------............         323
         32 M.SLFLLVGTL.I.FA.V....I.M....SAL.A...ER.TIN..A..S.S.------.....L.T..........S.........A.............................QGS.YP--------............         324
   V1.2  33 M.SLFLLVGTL.I.FA.V....I.M....SAL.AF..ER.TIN..A.NS.I.GST--S.....L.T..........R.........A.............................QGY.S---------W...........         325
         34 M.SLFLLVGTL.I.FA.V....I.M....SAL.A...ER.TIN..A..S.S.------.....L.T..........I.NT......A.............................Q.Y.SP--------W...........         326
         35 M.SLFLLVGTL...FV.V....I.M....SAL.A...ER.TIN..A..S.SIGST--SF....L.T..........S.........A.............................QGR.TP--------Y.......Q.A..         327
         36 M.SLFLLVGTL.I.FA.V....I.M....SAL.....ER.TIN..A..S.I.QSI--N.....L.T..........A.N.......A.............................QGKDTP--------L...........  C2     328
         37 M.SLFLLVGTL.I.SA.V....I.M....SAL.A...ER.TIN.QA..S.S.------.....L.T..........S.........A.............................QGY.S---------............         329
      ArL29 M.SLFLLVGTL.I.FV.V....I.M....SAL.A...ER.TIN..A..SINT------N.G..L.A...P.S....S.........A..A..........................QGS.FP--------L...........     AF128800
      ArL25 M.SLFLLVGTL.I.FA.V....I.M....SAL.A...ER.TIN..A..S.I.GST--S..T..L.T...I......R..V......A...............R.............QGRTTP--------W........A..        9436
      ArL33 M.SLFLLVGTL.I.FD.V....I.M....SAL.P...ER.TIF..A.NS.S.GTA--SF.A..L.T.R.P......A.N.......A.............................Q.INSP--------R...........        9437
         38 -.S..IH..CS.I.F.HD....-.L..S.SA.........TI...A..S.SN------W....L.T..........S..V.E....A.............................QGSSYP--------F...........     MG029330
         39 -.S..IH..CS.I.F.HD....-.L..S.SA.........TI...A..S.SN------W....L.T..........R.........A.............................Q.N.FP--------............         331
         40 -.S..IH..CS.I.F.HD....-.F..S.SA.........TIN..A..S.G.------S....L.T..........A.........A.............................Q.S.S---------W...........         332
   V1.3  41 -.S..IHF.CS.I.FTHD....-.L..S.SA.........TIN..A..S.Y.------S....L.T..........D.N.......V..........Y.................Q.Y.TP--------F.......T...         333
         42 -----------------D....-.L..S.SA...V.....TIN..A..S.G.------S....L.T..........DE........A.............................Q.S.IP--------L...........         334
         43 -----------------D....-.L..S.SA.........TI...A..S.SN------W....L.T...T......R.........A.............................QGTTVSRV---TAP............         335
         44 -----------------D....-.L..S.SI.........TI...A.NS.IN------W....L.T..KP......D.........A..G..........................Q.NMIP--------L...........         336
         45 -----------------D....-.L..S.SA.........TI.......S.TN------W....L.T...TP.....R.........A.............................Q.N.FP--------L...........         337
```

Supplementary figure 2. Alignment of sterlet IgL1 VL deduced amino acid sequences. The VL sequences fall into three subfamilies (V1.1-1.3, indicated at the left) and attached to either C1 or C2 constant domain (indicated at the right). Dots represent identical to consensus amino acid residues, dashes – gaps introduced for alignment. Asterisk denotes frameshift in V-J junction in the clone 05. The CDR regions were determined according to the IMGT standard and shaded in gray. Leader peptide (LP) boundaries are shown according to exon-intron structure of the genomic DNA.
