# Supplementary material for: Diversity of Immunoglobulin Light Chain Genes in Non-Teleost Ray-Finned Fish Uncovers IgL Subdivision into Five Ancient Isotypes
Source: Front Immunol. 2018 May 28;9:1079. doi: 10.3389/fimmu.2018.01079 (PMC5985310; doi:10.3389/fimmu.2018.01079)
Supplement: Supplementary file 8 [file data_sheet_3.PDF]

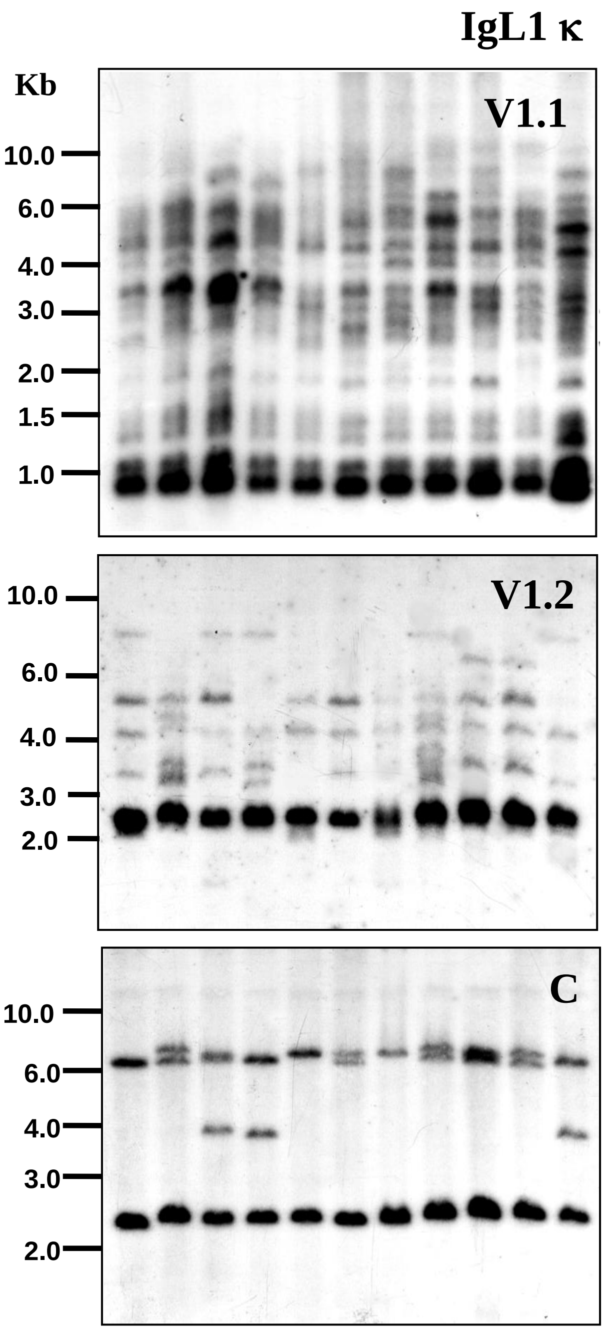

Supplementary figure 3. Southern blot analysis of sterlet genomic DNA. The blot was hybridized with either IgL1 V1.1-, V1.2- or C-specific probe and washed in strict conditions.
