# Supplementary material for: Diversity of Immunoglobulin Light Chain Genes in Non-Teleost Ray-Finned Fish Uncovers IgL Subdivision into Five Ancient Isotypes
Source: Front Immunol. 2018 May 28;9:1079. doi: 10.3389/fimmu.2018.01079 (PMC5985310; doi:10.3389/fimmu.2018.01079)
Supplement: Supplementary file 9 [file data_sheet_4.PDF]

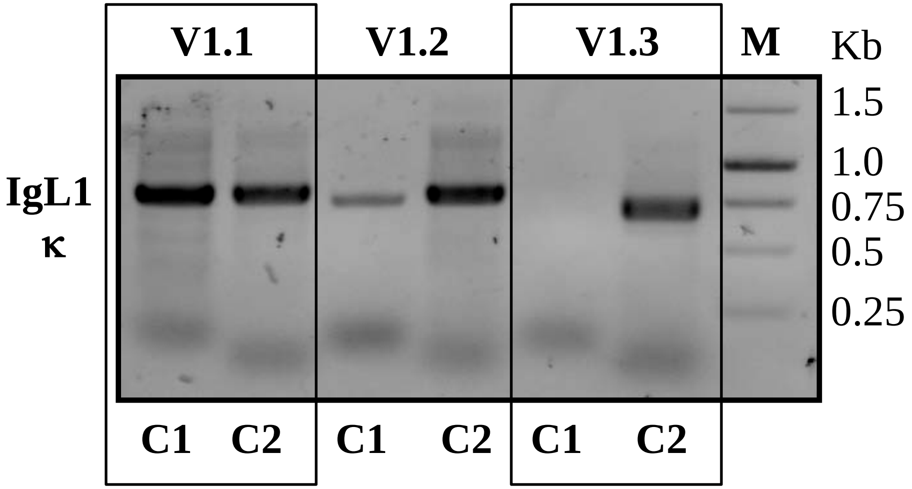

Supplementary figure 4. Cloning of Sterlet IgL1 cDNAs using RT-PCR. Forward primer specificity is indicated at the top, reverse primers – on the bottom of the gel image. Primer sequences may be found in the Supplementary table 2.
