# Supplementary material for: Diversity of Immunoglobulin Light Chain Genes in Non-Teleost Ray-Finned Fish Uncovers IgL Subdivision into Five Ancient Isotypes
Source: Front Immunol. 2018 May 28;9:1079. doi: 10.3389/fimmu.2018.01079 (PMC5985310; doi:10.3389/fimmu.2018.01079)
Supplement: Supplementary file 10 [file data_sheet_5.PDF]

```
IgL1 κ                                                     A

B1 B2 D Genomes         Germline Js      Scaffolds C1 C2
-  -  + GTACACTTTCGGACCGGGGACCAAGCTGGTTGTGAAAT J1  - +
-  +  + GTTCA..........G...................... J2  - +
-  +  + GTGG...........A...................... J3  + +
+  +  + GTAT...........G...................... J4  - +
+  +  + TCGT...........A...................... J5  - +
-  -  + GTAC..........AC...T..G............... J6  + -
+  +  + GTTG...........C............A......... J7  + -
+  +  + GTACA..........C...................... J8  + -
+  -  + GTAC...........C...T.................. J9  + -
        GTAC..........AC...T.................. J10 - -

V1.1 RSS-proximal end                                     B
GT--GTTCA                  Js from V1.1 cDNAs     #cDNAs
GT--GTTCA----..........G...................... J2      1
GT--GTT----G...........C............A......... J7    4
GT--GTT----G...........C........AT..A....A.... J7?   1
GT--GTT----G........G..C........A..AA......... J7?   1
GT--GT----AC...........G...................... J1    1+2
GT--GT----GG...........A...................... J3    3
GT--GT----AT...........G...................... J4      1
GT--GT----AC...........C...................... J8    9
GT--GT----GT...........A...................... J3?   1
GT--GT----AC...........C.........T............ J9?   1
GTGTT--CA----..........C.............A........ J8?   1
GTGTT--CA----..........C...................... J8    2
GTGTTC--A--............C...................... J8    1*
G----TA----............C...................... J8    1
GT--gGC---TC...........A...................... ??    1
                                                     32
V1.2/V1.3 RSS-proximal end                                C
CC--TCTCA                Js from V1.2/V1.3 cDNAs  #cDNAs
CCTC--TCA----..........G...................... J2      3
CCTC--TCA----..........G........A............. J2?     2
CCTt--TCA----..........G...................... J2      1
CC--T-----AC...........G...................... J1      2
CC--T-----GG...........A...................... J3    1+2
CC--T-----AT...........G...................... J4      1
C----CG--TAC...........G.....TC......C........ J1?     1
C----CG--TAC...........G...................... J1      3
C----CG--TTC...........G........AAC........... J2?     1
C----CG--TGG...........A...................... J3    1+1
C----CG--TGG...........A.............C.G...... J3?     1
CCT--CG----G...........A...................... J3      1
                                                     21
```

Supplementary figure 5. V-J junctions from cloned IgL1 cDNAs. Germ-line J1-10 sequences were originated from B1, B2, and/or D genomes (A). V1.1-J junctions (B) usually code for Valine residue, V1.2/1.3 junctions (C) code for Proline or contain gap instead (---). Shared nucleotides found in both V and J segments are gray-shaded. P-nucleotides are italicized. C1 scaffold-specific J segments with Cytosineare marked by green; C2 specific Js with Guanine – by blue; J3 segment with Adenosine present in both C1 and C2 scaffolds – by yellow. The number of cloned IgL cDNA with C1 (green) or C2 (blue) is shown at the right. Asterisk denotes cDNA with frameshift in the V-J junction.
