# Supplementary material for: Diversity of Immunoglobulin Light Chain Genes in Non-Teleost Ray-Finned Fish Uncovers IgL Subdivision into Five Ancient Isotypes
Source: Front Immunol. 2018 May 28;9:1079. doi: 10.3389/fimmu.2018.01079 (PMC5985310; doi:10.3389/fimmu.2018.01079)
Supplement: Supplementary file 11 [file data_sheet_6.PDF]

```
IgL2 κ
              V1          F                                      J1                            VJ junction
 Y  H  C  G  Q  S  D  A  L  P  F             Y  T  F  G  Q  G  T  R  L  I  V  K
TACCACTGTGGGCAGAGTGATGCAYTCCCTTTCA-RSS + RSS-GTACACTTTCGGCCAGGGGACCAGGCTGATTGTAAAGAgt
............................          CG      TAC.....................................   >  YHCGQSDAXPYTFGQGTRLIVK  Y
.............................TTT      CA         .................................   >  ..........F...........  F
.............................      TTTC               ...........................   >  ..........--..........  --
              V2                                                 J1
 Y  Y  C  M  Q  S  D  T  F  P  F             Y  T  F  G  Q  G  T  R  L  I  V  K
TACTACTGTATGCAGAGTGATACATTCCCTTTCA-RSS + RSS-GTACACTTTCGGCCAGGGGACCAGGCTGATTGTAAAGAgt
............................          CG      TAC.....................................   >  YYCMQSDTFPYTFGQGTRLIVK  Y
.............................TTT      CA         .................................   >  ..........F...........  F
              V3
 Y  F  C  Q  Q  D  Y  S  T  P                Y  T  F  G  Q  G  T  R  L  I  V  K  J1
TACTTTTGTCAGCAGGACTATAGCACCCCTTT-RSS  +  RSS-GTACACTTTCGGCCAGGGGACCAGGCTGATTGTAAAGAgt
............................      CTTT                ..............................    >  YFCQQDYSTP--FGQGTRLIVK  --
.............................      T           ...................................    >  ..........TLSARGPG*      *
.............................                    .................................    >  ..........LSARGPG*       *
                                             W  T  F  G  Q  G  T  K  V  V  V  K  J2
                                        RSS-TTGGACATTCGGGCAAGGGACCAAGGTGGTGGTAAAAAgt
............................       C              ...............................    >  ..........--.....KVV..  --

IgL3 λ
              V                                                    J
 Y  C  G  V  W  H  S  G  S  N  R  F                F  V  F  G  A  G  T  A  L  H  A  S
TATTGTGGTGTGTGGCACTCAGGCTCAAACAGATTCA-RSS + RSS-CATTCGTCTTCGGGGCAGGGACCGCACTGCACGCCAGCAgt
................................        ATTC        ..................................  > YYCGVWHSGSNRFVFGAGTALHASS
```

Supplementary figure 6. V-J junctions from cloned IgL2 and IgL3 cDNAs. IgL2 V-J junctions either code for Tyrosine (Y) or Phenylalanine (F) amino acid residues or contains double gap (--). Some cDNAs have frame shift in the junction (*). IgL3 junctions always code for Phenylalanine (F). Nucleotides found in both V and J segments are gray-shaded. P-nucleotides are italicized.
