# Supplementary material for: Diversity of Immunoglobulin Light Chain Genes in Non-Teleost Ray-Finned Fish Uncovers IgL Subdivision into Five Ancient Isotypes
Source: Front Immunol. 2018 May 28;9:1079. doi: 10.3389/fimmu.2018.01079 (PMC5985310; doi:10.3389/fimmu.2018.01079)
Supplement: Supplementary file 12 [file data_sheet_7.PDF]

Acipenceridae IgL sequences

IgL1 kappa

Polyodon spathula C1631680 26.0|RSZYR13120571_A-Polyodon_spathula Gonad IgL1 LP+V1.1

AAGACACTTTGCATTGGCAGCACATGCTTCAGTGCTCTGGGAGTGTTTATAGCCCTGTGAGTTTAACACTTCATGAT
TGAGAGCTGCCCTTCATGGCAACAGAATCCACAACAACAATGACTTTTATCAGCATCTTCATCTGGGCACTTGTCAT
CTGCACTCAGGAATCCAGTGGACAGTATACTGTGACTCAGACTCCAGCAGTGAAATCTGTTCTCCCAGGAGACACAG
TCGCTCTGAGCTGTAAAGTCAGCAGCGCAGTGTACAGT

MTFISIFIWALVICTQESSGQYTVTQTPAVKSVLPGDTVALSCKVSSAVYS

Polyodon spathula C1517372 5.0|RSZYR13120575_A-Polyodon_spathula Gill IgL1 LP+V1.2

ACCTTTCAGAGCTCCTGAAACACACAATAAAATGATGTCCTTGTTTCTCCTGGTTGGGACGCTTCTCATCATCTTTG
CCCAGGTCTCCAGTGGGCAGATCACTATGACTCAGACTCCTTCAGCGCTCTCTGCTCTCCCAGGAGAACGAGTCTCT
ATCAGCTGTAAAGCCAGCAGCTCCCTTGACAGCAGTGGTAAAAGCTACCTAGCCTGGTACCTGCAGACACCTGGAGA
AGCCCCTAAACTCCTGATCTATCTAGCAAGCACCCTTCAATCTG

MMSLFLLVGTLLIIFAQVSSGQITMTQTPSALSALPGERVSISCKASSSLDSSGKSYLAWYLQTPGEAPKLLIYLAS
TLQS

Polyodon spathula C983664 55.0|RSZYR13120573_A-Polyodon_spathula Liver IgL1 LP+V1.3

CAGCCCTTCAGTCAACACAATCTGAAACAACAATGAGTTTTATAATTCCCTTCATCTGCTCGCTGATAATCTTCACT
CATGATTCCAGTGGACAGACTTTGACTCAGTCTCCATCAGCTAAATCTGTTCTCCCTGGAGATACTGTCACTATCAA
CTGCAAAGCCAGCAGCTCTGTGAGCAGCTACCTAAACTGGTACCTGCAGACACCTGGAGAA

MSFIIPFICSLIIFTHDSSGQTLTQSPSAKSVLPGDTVTINCKASSSVSSYLNWYLQTPGE

Polyodon spathula scaffold22318 Locus_40014_1 58.1 COMPLEX|RSZYR13120573_A-Polyodon_spathula Liver IgL1 J+Ca

GTGGACTTTCGGGCCCGGGACCAagctgcttgtgaaatctggaagccccattgctccttcctccgtctccgtgATCC
CTCCCTCCATGCTGGAGCTCAACACAAAGAACAAAGCCACCCTGGTCTGCCTGGTGAATAATTTCTACCCCGATGCC
GTGGATATCAAGTGGAAAGTGGACGAGGTGGTCCAGACCAGTGGTGTGCTGACCAGCTCAATGAAGCAGAAGGACGG
CAAATACAGCGCAAGCAGCAGCTTGACCCTGACCAAGGCCCAGTGGGACCAGAAGGAGAAGTACACCTGCGTTGTGA
CCCACGAGGCCGTGAGCACTCCAATGAGCGAGACCATCTACAAGAGCCAGTGCACACTGTTGGATGCCTAG

WTFGPGTKLLVKSGSPIAPSSVSVIPPSMLELNTKNKATLVCLVNNFYPDAVDIKWKVDEVVQTSGVLTSSMKQKDG
KYSASSSLTLTKAQWDQKEKYTCVVTHEAVSTPMSETIYKSQCTLLDA*

Polyodon spathula scaffold20456 Locus_35718_0 44.0 COMPLEX|RSZYR13120573_A-Polyodon_spathula liver IgL1 J+Cb

TCGTACTTTCGGACCAGGGACCaagctggttgtgagatctggaagcccCATTGCTCCTTCCTCCGTCTCCGTGCTGC
CTCCCTCCCAGCTGGAGCTCAACACAAACAAAGCCACCGTGGTCTGCATGGTGAATAATTTCTACCCCGATGTCGTG
GCTATCAAGTGGCAAGTGGACGGGGTGGACCAGTCGAATGGTGTGCTGACCAGCTCAGTGAAGCAGAAGGACGGCAA

ATACAGCGCAAGCAGCAGCCTGACCCTGACCAAGGCCCAGTGGAACCAGCAGGAGAAGTACACCTGCATTGTGACCC
ACGAGGCCATGAGCACTCCGATGAGCGAGACCATCAGCAGGAGCCAGTGCACACTGTTGGATGCC

RTFGPGTKLVVRSGSPIAPSSVSVLPPSQLELNTNKATVVCMVNNFYPDVVAIKWQVDGVDQSNGVLTSSVKQKDGK
YSASSSLTLTKAQWNQQEKYTCIVTHEAMSTPMSETISRSQCTLLDA

Acipenser sinensis gb|GETX01015768.1| TSA: Acipenser sinensis
comp109755_c0_seq1 transcribed RNA sequence IgL1 V1.1 complete

ATGACTTTTATCAGCATCTTCATCTGGGCACTTGTCATCTGCACTCAGGAATCCAGTGGACAGTATACTGTGACTCA
GACTCCAGCAGTGAAATCTGTTCTCCCAGGAGACACAGTCGCTCTGAGCTGTAAAGTCAGCAGCGCAGTGTACAGTA
ACAACTACCTAGCCTGGTACCAACAGAAACCTGGAGAAGCTCCCAAACTCCTGATCTATGCTGCAAGTACCCTTCAG
TCTGGGATCCCAACTCGTTTCAGTGGCAGTGGATCTGGGACTGACTTCACTCTGACCATCAGTGGAGTCCAGGCTGA
AGATGCAGGAGATTACTACTGTCAGAGTTTCCACTACCCCAGCAGTAGATATGTGTACACTTTCGGACCCGGGACCA
AGCTGATTGTGAAATCTGGAAGCCCAACTGCTCCTTCCTCCGTCTCCCTGCTTCCTCCCTCCAAGGTGGAGCTCGAC
ACAAAGGGCAAAGCCACCTTGGTCTGCCTGGTGAATAATTTCTACCCCGATGTCGTGGATATCAAGTGGACGGTGGA
CGGGGTGGCCCAGTCGACTGGTGTTCTGACCAGCACAATGAAGCAGAAGGACGGGAAATACAGCGCAAGCAGCAGCC
TGACCCTCACCAAGGCCGTGTGGAACAGCAAGGAGAAGTACACCTGCACTGTGAAGCACGAGGCTGTGAGCACTCCC
AGGAGCGAGTCCATCAACAGGAGCGAGTGCACACTATTAGATGCCTAA

MTFISIFIWALVICTQESSGQYTVTQTPAVKSVLPGDTVALSCKVSSAVYSNNYLAWYQQKPGEAPKLLIYAASTLQ
SGIPTRFSGSGSGTDFTLTISGVQAEDAGDYYCQSFHYPSSRYVYTFGPGTKLIVKSGSPTAPSSVSLLPPSKVELD
TKGKATLVCLVNNFYPDVVDIKWTVDGVAQSTGVLTSTMKQKDGKYSASSSLTLTKAVWNSKEKYTCTVKHEAVSTP
RSESINRSECTLLDA*

Acipenser sinensis gb|GETX01007387.1| TSA: Acipenser sinensis
comp107139_c0_seq2 transcribed RNA sequence IgL1 LP+V1.2

ATGATGTCCTTGTTTCTCCTGGTTGGGACGCTTCTCATCATCTTTGCCCAGGTCTCCAGTGGGCAGATCACTATGAC
TCAGACTCCTTCAGCGCTCTCTGCTCTCCCAGGAGAAAGAGTCACTATCAACTGCAAAGCCAGCAGCTCTGTTAGCA
GTGGTAGTACAAGCTACCTAGCCTGGTACCTGCAGACACCTGGAGAAGCTCCTAAACTCCTGATCTATAGTGCAAGC
ACCCTTCAATCTGGGATCCCAGCTCGTTTCAGTGGCAGTGGATCT

MMSLFLLVGTLLIIFAQVSSGQITMTQTPSALSALPGERVTINCKASSSVSSGSTSYLAWYLQTPGEAPKLLIYSAS
TLQSGIPARFSGSGS

IgL2 kappa

Acipenser sinensis gb|GETX01009563.1| TSA: Acipenser sinensis
comp108891_c0_seq1 transcribed RNA sequence IgL2 LP+V1

ATGATCTCACTCAGTGTTCTGGTCTGTTTACTGATTGTCTGGACAAAGGATTCCAGAGGTGACATTACAGTAGATCA
GTCACCCCCTGCCATATCAGTTGTGCCAGGTCAAACTGTCACTATAAAGTGTAAAGCCTCCCAGGTTATTGGTGATG
ATATGGAGCTGTATCAGTTTAAACCTGGACATGCACCTAAGATGTTAATATATGATGGAAATAAACTTTTCACTGGA
ACCCCAAGTCGGTTCAGTGGTACCTGGAGTGGAGCTGACCACACGTTCACCATCAGCAATGTCCAGAGTGAAGACGA
TGCAGAATACCACTGTGGGCACAGTGATGCACTCCCTTT

MISLSVLVCLLIVWTKDSRGDITVDQSPPAISVVPGQTVTIKCKASQVIGDDMELYQFKPGHAPKMLIYDGNKLFTG
TPSRFSGTWSGADHTFTISNVQSEDDAEYHCGHSDALP

Acipenser sinensis gb|GETX01016326.1| TSA: Acipenser sinensis comp110182_c1_seq4 transcribed RNA sequence IgL2 J+C

GTACACTTTTGGCCAGGGGACCAGGCTGATTGTAAAGAGCCGAGCGCTGGCTTCCCCGGTAGTCAGTCTGCTACCTC
CTTCAACTGAAGAGCTCTCAAAGAACCGGGCAACGCTTGTGTGCCTGGTCGACAAATTCTACCCAGACATCGTCGAA
GTCGTGTGGGAAATCGACGACAAGAGCCAGACCGGCGGGATTCTCAACAGCAAGTCGCTCAAGGCGTCAGACAACAC
GTACAGCATGAGCAGCATCCTCACGCTGACGCGGGCAAAGTGGGAGTCCAGCGAAAAATACTCCTGCATCATCAAGC
ATGAAAACTCCGCAGCCCCTATTGTGAGCACCATCAATAGAAGCCAATGTACAACTGCGTAA

YTFGQGTRLIVKSRALASPVVSLLPPSTEELSKNRATLVCLVDKFYPDIVEVVWEIDDKSQTGGILNSKSLKASDNT
YSMSSILTLTRAKWESSEKYSCIIKHENSAAPIVSTINRSQCTTA*

Polyodon spathula C1786232 19.0|RSZYR13120574_A-Polyodon_spathula Brain IgL2 J+C

GCCCAGACAGCTAAAAGCACTAACATGTTAGAACGGTATACAGCTATGTAGCCTACTACTGTATTTATATCAATATT
TAAACCAGTATCTTGAAACAATGGCAATGGTTTGACTTCTCAGCTACTATTACACAGTTGTACATTGGCTTCTATTG
ATGGTGTTCTCAATAGGGGTTGCGGAGTTTTCATGCTTGACGGTGCATGAGTATTTTTCGCCGGACTCCCACTTTGC
CCGTGTCAGAGTGAGGATGCTGCTCATGCTGTACGTGTTGTCTGACGCCTTGAGGGATTTGCTTTTGAGAACCCCGT
CGGTCTGTCCCTTGTCGTCGATTTTCCACACGACTTCGACGATGTCTGGGTGGAATTTGTCGACCAGGCACACAAGC
GCTGCCCGGTTCTTTGCGAGCTCTTCAGTTGGAGGAGGAAGCAGACTGACTACCGGTGAAGCCAGCGCTCGGCTCTT
TACAATTATCCTGGTCCCCTGGCCAAAGGTGTACCACAGTGATCAGTTCAGAACGACACACTGTTATCCTTTTTAAG
CAAATCCTGGATGTTTACTGCATTAAATGACTGGTTGCAAGTTCCA

SLWYTFGQGTRIIVKSRALASPVVSLLPPPTEELAKNRAALVCLVDKFHPDIVEVVWKIDDKGQTDGVLKSKSLKAS
DNTYSMSSILTLTRAKWESGEKYSCTVKHENSATPIENTINRSQCTTV

Acipenser baerii TRINITY_DN79864_c6_g1_i1 IgL2 V1 complete

ATGATCTCACTCAGTGTTCTGGTCTGTTTACTGATTGTCTGGACAAAGGATTCCAGAGGTGACATTACAGTAGATCA
GTCGCCCCCTGCCATATCAGTTGTGCCAGGTCAAACTGTCACTATAAAGTGTAAAGCCTCCCAGGTTATTGGTGATG
ATATGGAGCTGTATCAGCTTAAACCTGGACATGCACCTAAGATGTTAATATATGATGGAAATAAACTTTTCACTGGA
ACCCCAAGTCGGTTCAGTGGTACCTGGAGTGGAGCTGACCACACGTTCACCATCAGCAATGTCCAGAGTGAAGACGA
TGCAGAATACCACTGTGGGCAGAGTGATACAAGCCCTTTCACTTTCGGCCAGGGGACCAAGCTGATTGTAAAGAGCC
GAGCGCTGGCTTCCCCGGTTGTCAGTCTGCTACCTCCTTCAACTGAAGAGCTCTCAAAGAACCGGGCAACGCTTGTG
TGCCTGGTCGACAAATTCTACCCAGACATCGTCGAAGTCGTGTGGGAAATCGACGACAAGGGCCAGACAGACGGGAT
TCTCAACAGCAAGTCGCTCAAGGCGTCAGACAACACGTACAGCATGAGCAGCATCCTCACGCTGACGCGGGCAAAGT
GGGAGTCCAGCGAAAAATACTCCTGCATCATCAAGCATGAAAACTCCGCAGCCCCTATTGTGAGCACCATCAATAGA
AGCCAGTGTACAACTGCGTAA

MISLSVLVCLLIVWTKDSRGDITVDQSPPAISVVPGQTVTIKCKASQVIGDDMELYQLKPGHAPKMLIYDGNKLFTG
TPSRFSGTWSGADHTFTISNVQSEDDAEYHCGQSDTSPFTFGQGTKLIVKSRALASPVVSLLPPSTEELSKNRATLV
CLVDKFYPDIVEVVWEIDDKGQTDGILNSKSLKASDNTYSMSSILTLTRAKWESSEKYSCIIKHENSAAPIVSTINR
SQCTTA*

Acipenser baerii TRINITY_DN80040_c0_g1_i1 IgL2 LP+V2+J

ATGATGTCACTTAACTATTTGGTCTTTTTACTGATCACCTTGGTTCAAGATTCCAGGGCTGACATTACTGTAGATCA
GTCCCCTGTTGCTATTTCTGTGAATCAAGGGGAAACTGTCACTATTAAGTGCAAAGTCTCAACACAAATTGATAGAG
ATATGGAGTTGTTTCAACAGAAACCAGGACAAGAACCCAAGCTCCTTGTTTATAATTCAGATACTCTTTTCACTGGG
GTCTCCTCTCGGTTCAGTGGTACTTATAGTGGCACTGACCACACATTTACTGTCAGCAATATCCAGCATGAAGACAA
AGCAGAATACTACTGTATGCAGAGTGATACATTCCCTTTCACTTTCGGCCAGGGGACCAAGCTGATTGTAAAGAGC

MMSLNYLVFLLITLVQDSRADITVDQSPVAISVNQGETVTIKCKVSTQIDRDMELFQQKPGQEPKLLVYNSDTLFTG
VSSRFSGTYSGTDHTFTVSNIQHEDKAEYYCMQSDTFPFTFGQGTKLIVKS

Acipenser baerii TRINITY_DN74717_c0_g1_i1 IgL2 LP+V3

ATGTCAATGATTAGCTTGCTGGCTACCCTGTTAATCTTAATCCACGATCTGCATGGCCAGATTGTTATGGATCAGTC
TCCAGCTTCGTTGTCTGTTCTTAGAGGACAATCTGTCACTATCAAATGTAAAGCAGGGAGCTCTGTCAGCAGTGAGG
GACACTGGTATCAGTTTAAGGAAGGGGAAACTCCCAAGCTCCTGATTTATTGGACAAACAATCGTCATACTGGAGTC
CCAAGTCGGTTTAGTGGCTCCCAGTCTGGAACTGAATTAACTTTCACCATCAGCAATGTGCAGCCCGAGGATGATGG
AGATTACTTTTGTCAGCAGGACTATAGAACCCCTTT

MSMISLLATLLILIHDLHGQIVMDQSPASLSVLRGQSVTIKCKAGSSVSSEGHWYQFKEGETPKLLIYWTNNRHTGV
PSRFSGSQSGTELTFTISNVQPEDDGDYFCQQDYRTP

Acipenser ruthenus Miseq data predicted IgL2 LP+V2.1.6

ATGATCTCACTCAGTGTTCTGGTCTGTTTACTGATTGTCTGGACAAAGGATTCCAGAGGTGACATTACAGTAGATCA
GTCGCCCCCTGCCATATCAGTTGTGCCAGGTCAAACTGTCACTATAAAGTGTAAAGCCTCCCAGGTTATTGACGATG
ATATGGAACTGTACCAGCTTAAACCTGGACATGCACCTAAGATGTTAATATATGATGGAAATAAACTTTTCACTGGA
ACCCCAAGTCGGTTCAGTGGTACCTGGAGTGGAGCTGACCACACGTTCACCATCAGCAATGTCCAGAGTGAAGACGA
TGCAGAATACCACTGTGGGCAGAGTGATGCACTCCCN

MISLSVLVCLLIVWTKDSRGDITVDQSPPAISVVPGQTVTIKCKASQVIDDDMELYQLKPGHAPKMLIYDGNKLFTG
TPSRFSGTWSGADHTFTISNVQSEDDAEYHCGQSDALP

IgL3 lambda

Acipenser sinensis gb|GETX01090375.1| TSA: Acipenser sinensis
comp98716_c0_seq1 transcribed RNA sequence IgL3 J+C

CATTCGTCTTCGGGGCAGGGACCACACTGCACGCCAGCAGAACTCTGTCCACACCTTCACTGATCTTACTGGGAGCG
TCCTCTGATGAGCTGAAAGAAAAGAAACAGGCCACACTGGTGTGCCTGGTTGAGCATTTCTTCCCATATACAGTGAG
CGTGTCTTGGAAGGTGGATGGGACCGTAACAACCACCGGAGTCAAGACTGGAAAGCCGCAGCCACGAGCCGACAACA
GCTTCGATATGAGCAGCTACCTCACTCTGTCCGAGTCAGAATGGACCACGTACAAGAACTTCGCCTGTGAAGTCACC
CACCAGACGCTGCCCACTCCCGCCAGCAAATCCTTCAAGAAATCCGACTGCGCTTAA

FVFGAGTTLHASRTLSTPSLILLGASSDELKEKKQATLVCLVEHFFPYTVSVSWKVDGTVTTTGVKTGKPQPRADNS
FDMSSYLTLSESEWTTYKNFACEVTHQTLPTPASKSFKKSDCA*

Acipenser baerii TRINITY_DN76216_c1_g1_i1 Va IgL3 complete

ATGTTAACTCTGAGTTGTATCTGTGCTTTACTGGCCTGTATTGCAGGTGTCGGCGCCCAGCCTGTCTTGACTCAAAC
ATCGTCAGTGTCGGTTTCACCAGGACAGAGTGCCCAGATCCCCTGTACCATGAGCGATGGTTATGTCATCACTGGTT
ACTGGGTGAACTGGTACCAGCAGAAACATGGAAGCACCCCGAGATATCTTCTAAATTACAAATCCAACTCTGAAAAG
GACTCTGCAGCTCCTGCTCGCTTTTCTGCATCTAAAGACACTGCGGGCAATTCCTGCCATTTAATCATCTCCAGTGT
AGAAGCAGATGACTATGCTGAGTATTATTGTGGTGTGTGGCACTCAGGCTCAAACAGATTCGTCTTCGGGGCAGGGA
CCGCACTGCACGCCAGCAGAACTCTGTCCTCACCTTCACTGAGCTTACTGGGACCGACCTCTGATGAGCTGAAAGAA
AAGAAACTGGCCACACTGGTGTGCCTGGTTGAGCATTTCTTCCCATATACAGTGAGCGTGTCTTGGAAGGTGGACGG
GACCGTAAAGACCACCGGAGTCAAGACTGGGAAGCCGCAGCCACGAGCCGACAACAGCTTCGATATGAGCAGCTACC
TCACTCTGTCCGAGTCAGAATGGGCCACGTACAAGAACTTCGCCTGTGAAGTCACCCACCAGACGCTGCCCACTCCC
GCCAGCAAATCCTTCAAGAAATCCGACTGCGCTtaa

MLTLSCICALLACIAGVGAQPVLTQTSSVSVSPGQSAQIPCTMSDGYVITGYWVNWYQQKHGSTPRYLLNYKSNSEK
DSAAPARFSASKDTAGNSCHLIISSVEADDYAEYYCGVWHSGSNRFVFGAGTALHASRTLSSPSLSLLGPTSDELKE
KKLATLVCLVEHFFPYTVSVSWKVDGTVKTTGVKTGKPQPRADNSFDMSSYLTLSESEWATYKNFACEVTHQTLPTP
ASKSFKKSDCA*

Acipenser ruthenus D genome scaffold23972 IgL3 Vc

GTGTCGGAGCACCCACTCTGTCTTGACTCAGACATCGTCAGTGTCGGTTTCACCAGGACAGAGTGCCCAGATCCCCT
GTACCATGAGCGATGGTTATGTCATCACTGGTTACTGGGTGAACTGGTACCAGCAGAAACATGGAAGCACCCCGAGA
TATCTTCTACACTACAAATCCAACTCTGAAAAGGGCTCTGCAGCTCCTGCTCGCTTTTCTGCATCTAAAGACACTGC
AGGCAATGCCTGCCATTTAATCATTTCCAGTGTAGAAGCAGAGGACTATGCTGAGTATTATTGTGAAGTGTGGCACC
AAACTCAGGCAAATACA

CRSTHSVLTQTSSVSVSPGQSAQIPCTMSDGYVITGYWVNWYQQKHGSTPRYLLHYKSNSEKGSAAPARFSASKDTA
GNACHLIISSVEAEDYAEYYCEVWHQTQANT

Acipenser ruthenus D genome scaffold16338 IgL3 Vd

GTGTCGGAGTGCCCACTCTGTCTTGACTCAGACATCGCCAGGGTCGGTTTCACCAGAACAGAGTGCCCAGATCCCCT
GTACCATGAGCGATGGTTATGTCATCACTGGTTACTGGGTGAACTGGTACCAGCAGAAACATGGAAGCACCCCGAGA
TATCTTCTACACTACAAATCCAACTCTGAAAAGGGCTCTGCAGCTCCTGCTCGCTTTTCTGCATCTAAAGACACTGC
GGGCAATGCCTGCCATTTAATCATCTCCAGTGTAGAAGCAGAGGACTATGCTGAGTATTATTGTGAAGTGTGGCACC
CAAACTCAGGCAAATACA

CRSAHSVLTQTSPGSVSPEQSAQIPCTMSDGYVITGYWVNWYQQKHGSTPRYLLHYKSNSEKGSAAPARFSASKDTA
GNACHLIISSVEAEDYAEYYCEVWHPNSGKY

Polyodon spathula Fish T10K scaffold35388 Locus_80012_0 23.3
LINEAR|RSZYR13120574_A-Polyodon_spathula Brain IgL3 Vd and J+C

TGTCGGCGCCCAGCCTGTCTTGACTCAGACATCGTCAGTGTCGGTTTCACCAGAACAGAGTGCCCAGATCACCTGTA
GCATGAGTGATGGTGATGTCATCACTGGTTACTGGGTGAACTGGTACCAGCAGAAACATGAAAGCACCCCGAGCTAT
CTTCTACACTACAAATCCAACTCTGAAAAGGGCTCTGCAACTCCTGCTCGCTTTTCTGTATCAAAAGACACTGCTGG
CAATGCCTGTCATTTAACCATCTCCAGTGTAGAAGTAGGGGACTATGCTGAGTATTATTGTGAAGTGTGGCACCCAA
AATCAGGCAAACACA

VGAQPVLTQTSSVSVSPEQSAQITCSMSDGDVITGYWVNWYQQKHESTPSYLLHYKSNSEKGSATPARFSVSKDTAG
NACHLTISSVEVGDYAEYYCEVWHPKSGKH

CATTCGTCTTTGGGGTTGGGACCACACTGCACGCCAGCAGAACTCCGTCCTCACCTTCTTTGAGTTTACTGGGACCG
TCCTCTGATGAGCTGCAAGAGAAACGGGCCACATTGGTGTGCCTGGTTCAGCATTTCTTCCCATATGCACTGAGCGT
GTCTTGGAAGGTGGACGGGAGCGTAACTGCCGCCGGAGTCAAGACTGGGAAGCCGCAGCAGCGCGCTGACAACAGCT
TCGATATGAGCAGCTACCTCATTCTGTCCGAGTCGGAGTGGATCAAACACAAGAACTTCGCCTGTGAAGTCACACAC
CAGACGCTGTCCACCCCCGCCAGCAAATCCTTCAACAAATCCGACTGCTTTTAA

FVFGVGTTLHASRTPSSPSLSLLGPSSDELQEKRATLVCLVQHFFPYALSVSWKVDGSVTAAGVKTGKPQQRADNSF
DMSSYLILSESEWIKHKNFACEVTHQTLSTPASKSFNKSDCF*

IgL4 sigma

Acipenser ruthenus B1 TR142252|c0_g1_i1 LP+V-sigma

ATGAGGAAGATAGAGGGAGTGCGCTGCTGTTGTCTTGCAGTCTGGCTGAGCTGGGCTCTGTCTGTGACAGTGCTGAA
GGCTGCAGCCCGAAGCCTGTCTCTCAGCCCGGGACAGACAGCCGTCCTGGACTGCAGCATTGAGAGGGACAGCGGTG
AGCTTGTGAGCTGGTACAAGCAGAGCCCTGGGGATGCGCCTCAGTTTGTTCTCAGTCATTACCACACCAACAGCTCA
GCCGCTAACTACGGCTCCGGATTCTCCTCCGAGCGCTTCACTGCCGGGGCGAAGGACCCGCTCACCTACCAGCTAGT
GATTCGCAGTGTGGAGTGGAGCGATGTAGCCGTGTATTACTGTGGAGCATGGACTTCAGCACACGGATAA

MRKIEGVRCCCLAVWLSWALSVTVLKAAARSLSLSPGQTAVLDCSIERDSGELVSWYKQSPGDAPQFVLSHYHTNSS
AANYGSGFSSERFTAGAKDPLTYQLVIRSVEWSDVAVYYCGAWTSAHG*

Acipenser sinensis gb|GETX01007679.1| TSA: Acipenser sinensis
comp107413_c0_seq1 transcribed RNA sequence LP+V-sigma

ATGAGGAAGATAGAGGGAGTGCGCTGCTGTTGTCTTGCAGTCTGGCTGAGCTGGGCTCTGTCTGTGACAGTGCTGAA
GGCTGCAGCCCGCAGCCTGTCTCTCAGCCCGGGACAGACAGCCGTCCTGTACTGCAGCATTGAGAGGGACAGCGGTG
AGCTTGTGAGCTGGTACAAGCAGAGCCCTGGGGATGCGCCTCAGTTTGTTCTCAGCCATCACCACACCAACAGCTCA
GCCGCTAACTATGGCTCCGGATTCTCCTCCGAGCGCTTCACTGCCGGGGCGAAGGACCCGCTCACCTACCAGCTAGT
GATTCGCAGTGTGGAGTGGAGCGATGTAGCCGTGTATTACTGTGGAGCATGGACTTCAGCACACGGATAA

MRKIEGVRCCCLAVWLSWALSVTVLKAAARSLSLSPGQTAVLYCSIERDSGELVSWYKQSPGDAPQFVLSHHHTNSS
AANYGSGFSSERFTAGAKDPLTYQLVIRSVEWSDVAVYYCGAWTS*

Polyodon spathula Fish T10K C1802953 48.0|RSZYR13120574_A-Polyodon_spathula
LP+V-sigma

ATGTGGAAGATAGAGGGAGTGTGCTGCTGTTGTCTTGCAGTCTGGCTGAGCTGGGCTCTGTCTGCGACAGTGCTGAA
GGCTGCAGCTCGCAGCCTGTCTCTCAGCCCGGGACAGACAGCCGTCCTGGACTGCAGCATTGAGAGGGACAGCGGGG
AGTTCGTCAGCTGGTACAAGCAGAGCCCTGGGGATGCACCTGAGTTTGTCCTCAGCCACTACCACACCAACAGCTCA
GCCGCTAGCTACGGCTCCGGATTCTCCTCCGAGCGCTTCACTGCCAGGGCGAAGGACCCGCTCACCTACCAGCTAGT
GATTAGCAGTGTGGAGTGGAGCGACACAGCCGTGTATTTCTGTGGAGCATGGATTTAA

MWKIEGVCCCCLAVWLSWALSATVLKAAARSLSLSPGQTAVLDCSIERDSGEFVSWYKQSPGDAPEFVLSHYHTNSS
AASYGSGFSSERFTARAKDPLTYQLVISSVEWSDTAVYFCGAWI*

Acipenser oxyrinchus c145265_g1_i3 transcribed RNA sequence GEUL01022793.1
IgL4 complete

MRRTLWLWFWVAIWIRVLKGEEETRRGQYIFTQGTKLIVTAHQLPPPSVVVFQPSKEELSKGTATLVCLVSKLSGAL
VDISWTANETAVTSEVPSSRPSRESDGTFSVSSCLTVSAAEWREDRVYSCIVQQGASLTQRSIQQSRC*

Polyodon spathula Fish T10K Scaffold29622 Locus_61105_0 48.9
COMPLEX|RSZYR13120574_A-Polyodon_spathula Brain IgL4 complete
ATGAGGAGCACACTGTGGCTGTGGTTCTGGATTGCAGTCTGGATCAGTCCTAGGGTTTTGAAAGGAGAGGAGCGGAC
GAGAATGGGTCACTACATATTCACTCTGGGCACCAAGCTGGTAATAACAGCCCATCAGCTCCCTGCACCTTCAGTGG
TGGTCTTTCCACCTTCAGAAGAAGAGCTTGTCTCTAAAGGAACGGCCACTCTGGTTTGCCTGGTGAGCAAGCTGCCC
GGGGCTCTCGTTGGCATCAGCTGGACAGCCAATCAGATGGCAGTGACCAGCGAAGTCTTGCCCAGCCGGCCCTCCAG
GGAGTCTGACGGCACCTTCAGTGTGAGCAGCTGCCTGACCATCTCTGCGGCAGAGTGGAGGGAGGACAGAGTCTATT
CCTGTATCGTACAGCAGGGGGACTCGCTCACCCAGCGCTCCATACAACAATCTCACTGCTGA

MRSTLWLWFWIAVWISPRVLKGEERTRMGHYIFTLGTKLVITAHQLPAPSVVVFPPSEEELVSKGTATLVCLVSKLP
GALVGISWTANQMAVTSEVLPSRPSRESDGTFSVSSCLTISAAEWREDRVYSCIVQQGDSLTQRSIQQSHC*

Fish T1K database and Spotted gar genome IgL sequences

IgL kappa

Erpetoichthys calabaricus (Ropefish) FT10K Scaffold17368 Locus_100803_1 61.2 COMPLEX|140501185-Erpetoichthys_calabaricus Gill IgL complete

ATGAGCTCCCAGACTCTTCTTTTATGGGCAGCCATGCTCGTTTTTCAGGTTTGTACTGGTCAGATTGTTCTTGATCA
GCCTCCATCACAGTCCACAACTCCAGGACAGACCGTCTCTCTGAAATGTAAAGCCCGTACCTCTGTAGGGAGCTGTC
TGAACTGGTACCATCAGAGACCTGGCCAGGTACCCCGGCTCCTGATTTACTACGGCACCAACCGTCAATCTGGTGTT
CCAGACAGGTTCTCTGGCAGCTCTTCAGGGACAGATTTTAGCCTGACAATTAGTCGTTTTGAACCAGAAGATGCAGG
ATATTACTACTGTCAGCAGTGTAGCCAGTGGCCTTACACTTTCGGCGGTGGAACCAAGTTGACAGTGGGCTTGAAAG
ATAATGTGAAGCCAACAGTATCCGTGTTTCCTCCCTCTAAGGATGAACTCGAGGGCAACAAGAAGGCCACTCTGGCA
TGTCTGGTAAACAAGTTCTACCCGGAAGATTTAAAGGTGGAATGGTATAACGGTGAAAGCCTGATCTCTAGTGATGT
CAAGACAAGTGACACCTTACTGGAAACGGATGGAACCTTCAGCTTGAGCAGCACCGTCACCCTGACATACGACCAGT
GGAACTCCAAGAGCACTTTCTTCTGCAAAGTTACACATGTCACTCTGAGTCCATCACAGAAATGGCCTGTGTCGAGT
GCAGATTGTAGTGTTTAG

MSSQTLLLWAAMLVFQVCTGQIVLDQPPSQSTTPGQTVSLKCKARTSVGSCLNWYHQRPGQVPRLLIYYGTNRQSGV
PDRFSGSSSGTDFSLTISRFEPEDAGYYYCQQCSQWPYTFGGGTKLTVGLKDNVKPTVSVFPPSKDELEGNKKATLA
CLVNKFYPEDLKVEWYNGESLISSDVKTSDTLLETDGTFSLSSTVTLTYDQWNSKSTFFCKVTHVTLSPSQKWPVSS
ADCSV*

Polypterus endlicheri FT10K scaffold6460 Locus_12879_2 37.0 COMPLEX|WHYR15010138_A-Polypterus_endlicheri Gill V+J+C

CTGTTTGCTCAGAACAGCAGCGCCGACATTATCCTCAGTCAGCCCCAGCAGATCCAAACTGCTCTTGTAGGACAGAG
CGTCTCTCTCAAATGCACCACCAGCACCTCCATTAGTCAGTACTTGATGTGGTACCATCAGAAACCAGAGCAAGCTC
CCCGGCTCTTGATTTATGATGCTGTTAACCGCTTTACTGGGATTCCTGAGCGTTTCAGAGGCAGTGGATCAGGCACA
GACTTTACTCTAACCATCAGTGATGTCCAGGACGAAGATTCAGGATATTACTATTGTCAGCAGAGGCAAAGTTGGCC
CTTCACTTTCGGCGGTGGAACCAAGTTGACAGTGGCCTTGAAAAATAATGTGAAGCCTACAGTATCCGTGTTTCCTC
CCTCTAAGGATGAACTTGAGGGCGACAAGAAGGCCACTCTGGCATGTCTGGTAAACAAGTTCTACCCGGAAGATGTA
AAGGTGGAATGGTACAACGGTGACAGCCTGATCTCTGGTAATGTCAAGACAAGTGACACCTTACTGGAAACGGATGG
AACCTTCAGCTTGAGCAGCACCGTCACCCTGACAGCCGACCAGTGGAACTCCAAAAGCACTTTCTACTGCAAAGTTA
CACATGTCACTCTGGCTCCATCACAGAAATGGCCTGTGTCGAGTGCAGATTGTAGTGTTTAA

LFAQNSSADIILSQPQQIQTALVGQSVSLKCTTSTSISQYLMWYHQKPEQAPRLLIYDAVNRFTGIPERFRGSGSGT
DFTLTISDVQDEDSGYYYCQQRQSWPFTFGGGTKLTVALKNNVKPTVSVFPPSKDELEGDKKATLACLVNKFYPEDV
KVEWYNGDSLISGNVKTSDTLLETDGTFSLSSTVTLTADQWNSKSTFYCKVTHVTLAPSQKWPVSSADCSV*

Lepisosteus oculatus LG22 IgL V and C exons

TCAAGAGGTGACATTGTGGTCACTCAGTCTCCAACATTTCTGTCTGCTCCACTGGGAGGCACAGTGAAAATCAACTG
TAAATCTGTGACAGCGATAGATGATGACATGGCCTGGTACCACCAAAGACCTGGACAAGAACCTAAACTTCTGATTT
ATGAAGGTCCCAAACGCTTCACTGGAGTTCCTGACAGGTTCTCAGCATCTGGATATGACTTTGATTTCAATTTAACA
ATCAGCAACATCCAGGCAGAAGATGCAGGAGATTACTATTGTCAACAGCATGAAGCAAGACCTCTCA

SRGDIVVTQSPTFLSAPLGGTVKINCKSVTAIDDDMAWYHQRPGQEPKLLIYEGPKRFTGVPDRFSASGYDFDFNLT
ISNIQAEDAGDYYCQQHEARPL

GCAATGCTCAGCCCACACTAACAGTCCTCCCCCCTTCGAACACGGAGCTGTCGTCCAAGAAGACCGCCACGCTGCTT
TGCCTGGCCAACAAGGGCTTCCCAGCCAACTGGACCCTGCAATGGAAGGTGAATGGTGGCTCCCAGGGTTCCGGCGT
CACCAGAACCCCCGGGGTTCTAGACAGTGATGGCAAGTACAGCTGGAGCAGCACTCTGACACTGCCCCTGTCTGAAT
GGGACACGGCGACAACCGTAACCTGCGAAGCCACTCACAGCTCCCAGGCTACTGTCATGAAGGTCTTGAAGAAATCG
GAGTGCCTGTAA

NAQPTLTVLPPSNTELSSKKTATLLCLANKGFPANWTLQWKVNGGSQGSGVTRTPGVLDSDGKYSWSSTLTLPLSEW
DTATTVTCEATHSSQATVMKVLKKSECL*

IgL lambda

Lepisosteus oculatus LG5 IgL C pseudo exone

GGGGCCAGTTCTCCACCCTCCGTGAGCTTACTGATGCCCACACCAGCAGAGCAGGCTTCCCAGGATAGGAAGACTCT
CGTCTGCCTTGTGGACAGATTCTTCCCCAGAGCGGTGACGGTCTTGGAAAGCGGACGGGAGCGTATTAACGCGGGCG
CCCTGACAGCTTCTGCGGCGCAGCAGAGCGACAACAGCTTCAGCACCAGCAGCTGTCTGAGCCTGTCCGCCTCCGAC
TGGAGAGGACACGAAGCCTTCTCCTGCGAGGTGACCCATGAGGCGCTCTCCAGCCCCAAAGCCGTGTCCTTCAAGAA
ATCGGAATGCGTCTGA

GQFSPSVSLLMPTPAEQASQDRKTLVCLVDRFFPRAVTVLESGRERINAGALTASAAQQSDNSFSTSSCLSLSASDW
RGHEAFSCEVTHEALSSPKAVSFKKSECV*

IgL lambda-2

Polypterus endlicheri (Saddled bichir) FT10K Scaffold41524 Locus_108041_0
22.9 LINEAR|WHYR15010138_A-Polypterus_endlicheri Gill IgL V+J+C

TCCTGGACAGGCAGTCAGAATTTCCTGCACACCCAGTACAGGCACCTGGGTTATTACATGGTACCAGCAGAAGACTG
GTAGTGTACCCCGGTACTTACTATATGATTCAACTCGAGCATCAGGGCTTCCAGCACGGTTTAGAGCATCAGAGGAG
AATTCTGGAAGAATGGAATATCTTAATATTGATGATGTGCAGCTAGAAGATAATGCTGTCTACTACTGTGCATGCCA
TGGCTGTGATAGTTCTACTTTTTTTGGTGGAGGCACCAATCTGGAAGTGGGTAAAGCTACCACCCCACCTTTGATGA
CCTTGTTCCCCCCATCTAAAGAAGAGCTGTCCAGCACTGGCAAGGCCACCCTGCTTTGTATGGCCCAGGGCTTCTAT
CCTGGGTCTTTGAATGTTTTGTGGGCAGCAGGTGGCATTACCAAGACAGGGCGCGAGATCCAGACCAGTGAGGCTGA
GCAGCAGTCTGATGGCAGTTATCATTTCAGTAGTTTCTTACAGCTGAGTGCTGAAGAGTGGCAGTCTGGTCAGGAAT
TCTCCTGCCAATTGAGTCACCAAGCCCTGAGTTCCCCCATGAAGAAGAGTATCAGCAGCGCAAACTGTGCACAGTAG

PGQAVRISCTPSTGTWVITWYQQKTGSVPRYLLYDSTRASGLPARFRASEENSGRMEYLNIDDVQLEDNAVYYCACH
GCDSSTFFGGGTNLEVGKATTPPLMTLFPPSKEELSSTGKATLLCMAQGFYPGSLNVLWAAGGITKTGREIQTSEAE
QQSDGSYHFSSFLQLSAEEWQSGQEFSCQLSHQALSSPMKKSISSANCAQ*

Erythrinus erythrinus (Red wolf fish) FT10K S scaffold20976 Locus_42463_0
37.5 FORK|WHYR15010136_A-Erythrinus_erythrinus Gill V and J+C

V
GTCTGGACGCTCTGGTGTTGACGCAGGAGAAGACTCTTTCAGCTCAACTCGGCCAAAACCTGAAGATCCTGTGCTCC
TCTAGTAGCAGCAGGGATTGGACTTTAGCATGGTACCAGCAGAAACCTGGTGAAGTTCCAAAGTTCTTGCTTGCTGA
CAGCAACAGGGCAACTGGACTCCCAGCTCGATTCACTTACAGCGGATCTGGATCACAGGAATACCTGCACATTAACG
GAGTTCAGTCTGACGACGAGGCCGTTTACTACTGCGCTTGTCACGGCTGCGGTGGTTAT

LDALVLTQEKTLSAQLGQNLKILCSSSSSRDWTLAWYQQKPGEVPKFLLADSNRATGLPARFTYSGSGSQEYLHING
VQSDDEAVYYCACHGCGGY

J+C
TTTCTTTGGTGAAGGCACTGATGTAACTCTTGGTGGCAGTTCTTCTCCTCCGTCTCTGCTCCTCCTGGCCCCCCCTC
TGTCCGCCCCCTCTGGCTCTGAGGTCAGTGTGGTGTGTCTGGCTCAGGGCTTTCGACCCGATGCTGTGGTCGTGTCC
TGGACGGAGGACAGCAGCAGCATCGCGGGTCCCGAGGCCCAGAACGGACTCTCCCAGCGGCAAAAAGACGGCACTTT
CATCCAGAGCAGTGTCCTGAAACTCAGCCCAGAGCGCTGGAACTCTGGACACACCTTCACCTGCCAACTGACCCACC
CCTCCCTGAGCAGCCCCCTGAGAAAGAGCATCAGAGCGGGGCAGTGCAGCTAG

FFGEGTDVTLGGSSSPPSLLLLAPPLSAPSGSEVSVVCLAQGFRPDAVVVSWTEDSSSIAGPEAQNGLSQRQKDGTF
IQSSVLKLSPERWNSGHTFTCQLTHPSLSSPLRKSIRAGQCS*

IgL sigma

Erpetoichthys calabaricus Fish T10K C379220 28.0|140501187 -
Erpetoichthys_calabaricus Liver IgL complete

ATGTTGGCTGTCCTCTGCTTCCTGACATGCTGGCTGACAAATGCCATTGGTCAGAAGGTCCTGACTCAGCCGGGTGT
TATGTCTGTGAATCTGGGGGACACTGCCACACTAGACTGTAACATAGAGAAAGATGAGGGTAAATATGTCTTCTGGC
TGAAGCAGGTCCCAGGCAGTCCTCCCCAGCATATACTGGACTTCTATTATACTAAGAGTGCCTCTGAAGATTACGGT
ACTGGCTTCTCTTCCAGTCGGTTTAACTCGAAGGCCAAAAACAAAATTGATTATCAGTTACTAATCAGTAATGTAGA
GGCATCAGATAGTGCAGTGTATTACTGTCACACTTGGGACGACTCTGCCAGCAGCAGGGTATTCGGCAAAGGCACCA
AACTCATCGTTTCAGATGCCAACCTGCTGCCACCATCAGTCACCATCATCCCTCAGGTACCTGAGAATCTTGCAGAC
AGCGATACAGTAACCTTAGTCTGTATAGCCAATAAGCTGTCAGTCTCCCTCGCTGACCTCAAGTGGACATCAGATGG
CACAGAAGTGACCAGCAATGTGCAGACCAGTGACCCTGTGCAAGACTCGGACAAGACCTACAGCATTAGCAGCTACC
TGAGCATCACAGGCAGAGAGTGGAAGGCGGACAAAGTGTACGTCTGCACAGCGTCACAGGGCAGGTTGAGCTCCTCG
GCGTCCCAGCAGGTCCAGTATTCCAAGTGCAGTAAATAG

MLAVLCFLTCWLTNAIGQKVLTQPGVMSVNLGDTATLDCNIEKDEGKYVFWLKQVPGSPPQHILDFYYTKSASEDYG
TGFSSSRFNSKAKNKIDYQLLISNVEASDSAVYYCHTWDDSASSRVFGKGTKLIVSDANLLPPSVTIIPQVPENLAD
SDTVTLVCIANKLSVSLADLKWTSDGTEVTSNVQTSDPVQDSDKTYSISSYLSITGREWKADKVYVCTASQGRLSSS
ASQQVQYSKCSK*

IgL sigma-2

Erpetoichthys calabaricus Fish T10K scaffold8638 Erpetoichthys_calabaricus
Locus_58002_0 14.1 IgL complete

ATGAACCTCGTACAGATTCTGTGTACCATTTTATTACTGGGGGTTTATGTAAATGCTGGACCTGCTATTTTCTCTCA
GTCTTCATCAGTTATTCCTGTGACTCCTGGACAGACTGTCACCCTGGAATGTGCTGTGAAAAATGAAAATGTTTCAA
GAATTAACATGCTCTGGATTCGTCAGTCACCTGGAAAGGCCCCTGAAGGTGTCCTAACTTACAGAGCTGACAACAAA
ATCTACAGAGCTCCTGCGATTTCAGATCGATTTATACCTTCAAGAGATATAGTCAGGAGTTATCACCTTCTCACAAT
TAATAATGTCCAGGAAAATGATGACTCCAAATACTACTGTTTTATTTACTATGGTGATGGGACTCAAGCATGGGGCG
AAGGAACTCGCATACAAGTTCTGAAAAGTGACTTGTCTCCCCCATCTGTTCAAGTGTTTCGCACCCCAGAAGAACAA
CTCAGTGGTTCAGGTTATGTGACTCTCAGCTGCCTGGTGAGCGGTTTCTTCCCAGGGTACATAGACATCCAGTGGAC
TATGGATGGTCAGCAAGTGACAGACAACGTTCAGTCCAGCCCGGTGTCTTTGGACAGCAGTGGAAATTCCTTCTTGG
CCGTTAGTTACCTGAAGCTGCCCATTGCTAAGTGGAAGACTGATGTGAGATACTTCTGTATTGTCACTCATGAATCC
AGTAAGATGCCAGTCATTGGTTCTGTGGCACTTCAAGACTGCAATTCCTTC

MNLVQILCTILLLGVYVNAGPAIFSQSSSVIPVTPGQTVTLECAVKNENVSRINMLWIRQSPGKAPEGVLTYRADNK
IYRAPAISDRFIPSRDIVRSYHLLTINNVQENDDSKYYCFIYYGDGTQAWGEGTRIQVLKSDLSPPSVQVFRTPEEQ
LSGSGYVTLSCLVSGFFPGYIDIQWTMDGQQVTDNVQSSPVSLDSSGNSFLAVSYLKLPIAKWKTDVRYFCIVTHES
SKMPVIGSVALQDCNSF

Supplementary figure 7. Nucleotide and deduced amino acid sequences of fish IgL. Sequences are extracted from Acipenseridae genomic and transcriptomic datasets, Fish T1K transcriptomic database, and L. oculatus genome (sections 2.9 and 2.10).
