# Supplementary material for: Diversity of Immunoglobulin Light Chain Genes in Non-Teleost Ray-Finned Fish Uncovers IgL Subdivision into Five Ancient Isotypes
Source: Front Immunol. 2018 May 28;9:1079. doi: 10.3389/fimmu.2018.01079 (PMC5985310; doi:10.3389/fimmu.2018.01079)
Supplement: Supplementary file 14 [file data_sheet_9.PDF]

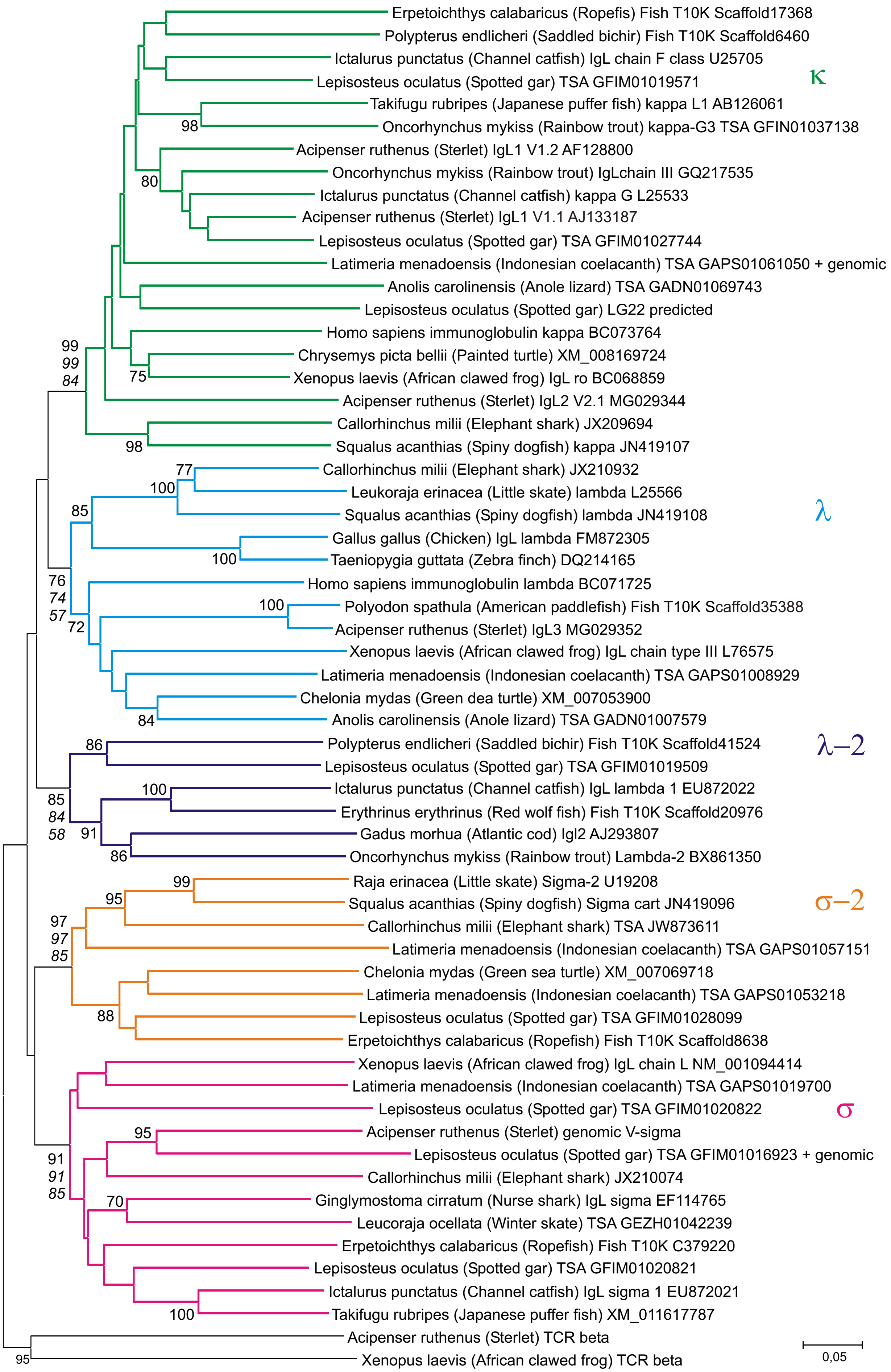

Supplementary figure 9. Phylogenetic analysis of VL sequences. The tree was constructed by the Neighbor-joining (NJ) method using nucleotide sequences after amino acid alignment. The bootstrap test values (500 replicates) equal or higher 70% are only shown. Maximum Likelihood (ML) and Minimum Evolution (ME) trees were essentially the same as the NJ tree in the major branching patterns. ML and ME bootstrap values are shown below NJ bootsraps and italicized.
