# Supplementary material for: Diversity of Immunoglobulin Light Chain Genes in Non-Teleost Ray-Finned Fish Uncovers IgL Subdivision into Five Ancient Isotypes
Source: Front Immunol. 2018 May 28;9:1079. doi: 10.3389/fimmu.2018.01079 (PMC5985310; doi:10.3389/fimmu.2018.01079)
Supplement: Supplementary file 15 [file data_sheet_10.PDF]

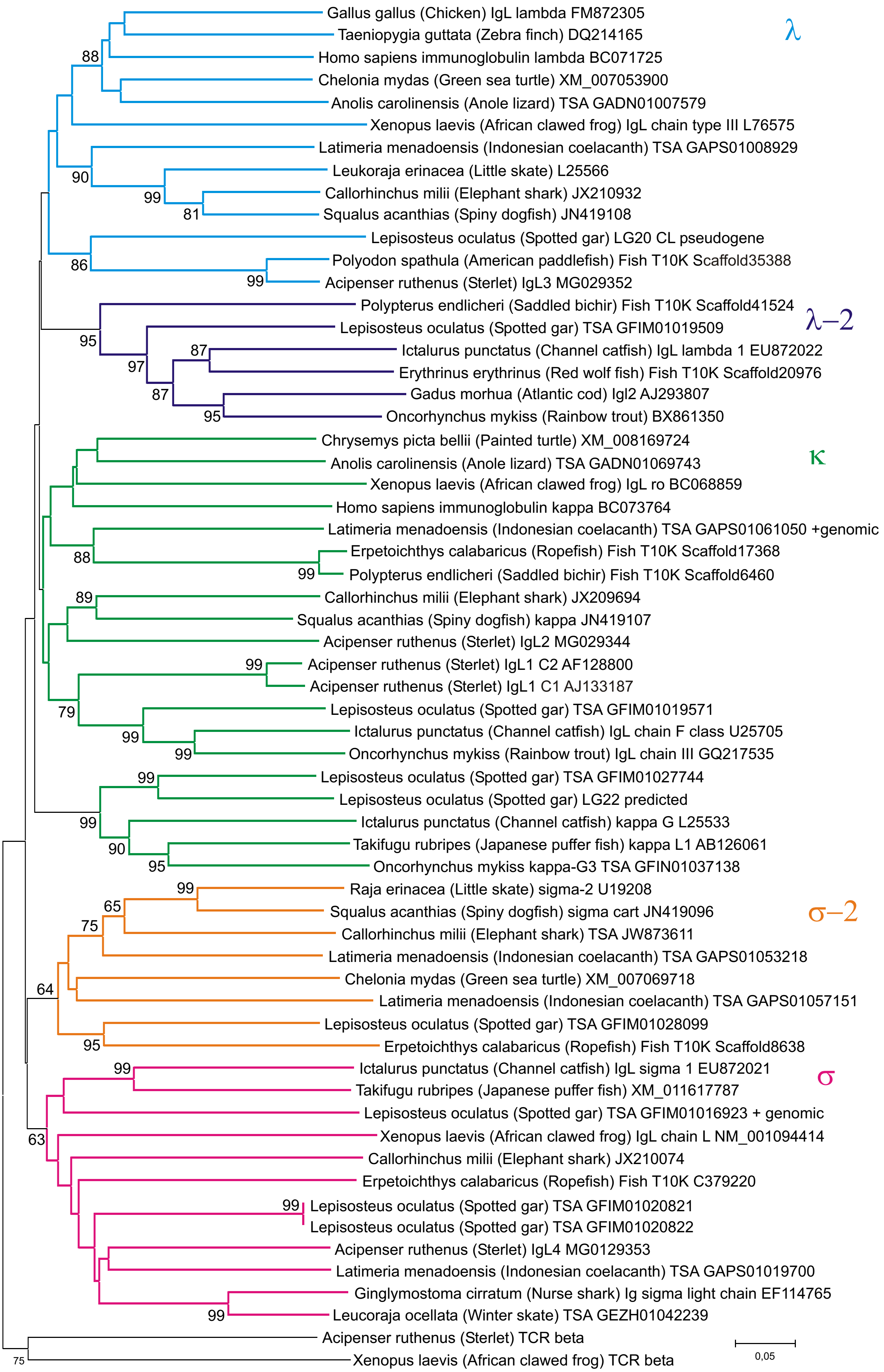

Supplementary figure 10. Phylogenetic analysis of CLsequences. The tree was constructed by the Neighbor-joining (NJ) method using nucleotide sequences after amino acid alignment. The bootstrap test values (500 replicates) equal or higher 70% are only shown. Maximum Likelihood (ML) and Minimum Evolution (ME) trees were essentially the same as the NJ tree in the major branching patterns.
